# Holotomography and atomic force microscopy: a powerful combination to enhance cancer, microbiology and nanotoxicology research

**DOI:** 10.1186/s11671-024-04003-x

**Published:** 2024-04-09

**Authors:** Iliana E. Medina-Ramirez, J. E. Macias-Diaz, David Masuoka-Ito, Juan Antonio Zapien

**Affiliations:** 1 Department of Chemistry, Universidad Autónoma de Aguascalientes, Av. Universidad 940, Aguascalientes, Ags Mexico; 2 Department of Mathematics and Physics, Universidad Autónoma de Aguascalientes, Av. Universidad 940, Aguascalientes, Ags Mexico; 3 Department of Stomatology, Universidad Autónoma de Aguascalientes, Av. Universidad 940, Aguascalientes, Ags Mexico; 4 Department of Materials Science and Engineering, City University of Hong Kong, Kowloon, Hong Kong SAR People’s Republic of China

**Keywords:** Holotomographic microscopy (HTM), Atomic force microscopy (AFM), Lable-free, Nanomedicine, Nanotoxicology, Refractive index

## Abstract

Modern imaging strategies are paramount to studying living systems such as cells, bacteria, and fungi and their response to pathogens, toxicants, and nanomaterials (NMs) as modulated by exposure and environmental factors. The need to understand the processes and mechanisms of damage, healing, and cell survivability of living systems continues to motivate the development of alternative imaging strategies. Of particular interest is the use of label-free techniques (microscopy procedures that do not require sample staining) that minimize interference of biological processes by foreign marking substances and reduce intense light exposure and potential photo-toxicity effects. This review focuses on the synergic capabilities of atomic force microscopy (AFM) as a well-developed and robust imaging strategy with demonstrated applications to unravel intimate details in biomedical applications, with the label-free, fast, and enduring Holotomographic Microscopy (HTM) strategy. HTM is a technique that combines holography and tomography using a low intensity continuous illumination laser to investigate (quantitatively and non-invasively) cells, microorganisms, and thin tissue by generating three-dimensional (3D) images and monitoring in real-time inner morphological changes. We first review the operating principles that form the basis for the complementary details provided by these techniques regarding the surface and internal information provided by HTM and AFM, which are essential and complimentary for the development of several biomedical areas studying the interaction mechanisms of NMs with living organisms. First, AFM can provide superb resolution on surface morphology and biomechanical characterization. Second, the quantitative phase capabilities of HTM enable superb modeling and quantification of the volume, surface area, protein content, and mass density of the main components of cells and microorganisms, including the morphology of cells in microbiological systems. These capabilities result from directly quantifying refractive index changes without requiring fluorescent markers or chemicals. As such, HTM is ideal for long-term monitoring of living organisms in conditions close to their natural settings. We present a case-based review of the principal uses of both techniques and their essential contributions to nanomedicine and nanotoxicology (study of the harmful effects of NMs in living organisms), emphasizing cancer and infectious disease control. The synergic impact of the sequential use of these complementary strategies provides a clear drive for adopting these techniques as interdependent fundamental tools.

**Graphical abstract:**

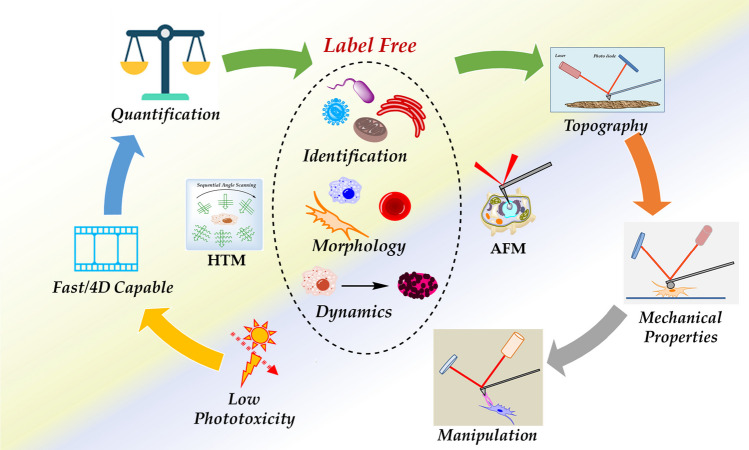

## Introduction

Nowadays, studying cell biology requires multiple procedures, chemicals, and imaging equipment for retrieving essential information on cell biology and biochemistry. Imaging methods range from complexity and cost depending on their ability to recover specific data [1, 2]. For example, light microscopy is widely used. Still, it only shows a low-resolution 2D perspective of cells and microorganisms. On the other hand, electron microscopies (i.e., scanning electron microscopy, SEM) render a more detailed view of the cell morphology [3, 4]. However, this technique requires sample fixation and a conductive cover for imaging and is not suitable for analyzing living cells [3].

Furthermore, confocal microscopy allows the distinction of cell organelles through fluorescent marker staining [1]. However, fluorescent molecules can cause a certain degree of phototoxicity, photobleaching, and intervention in cell mechanisms due to fluorescent markers aggregation, not to mention their high-cost [5, 6]. New developments in imaging techniques are required in biological sciences to access precious information without requiring complicated sample preparation procedures or reagents that can alter different ranges of cell biology [7]. While numerous alternatives have been developed in recent years for live-cell imaging, scientists still face difficulties when using modern light microscopy as, more often than not, a number of considerations must be made to balance sample integrity (photo-damage), acquisition speed (to visualize biomolecular events), signal-to-noise, and spatial resolution. A recent study compares the different super-resolution microscopy techniques, remarking their current advantages and limitations [1].

Techniques such as HTM rely on the interaction of light with different media within cells. HTM (also known as Optical Diffraction Tomography, ODT) is a versatile alternative for cell imaging that overcomes the disadvantages that the techniques mentioned above have [3]. HTM pieces of equipment make use of refractive index (RI) for cell investigation at fast analysis rates [3], including the possibility of adopting other techniques such as fluorescence for more precise identification of the molecular composition present in the cells [7, 8]. Besides the visualization of cells in 3D, HTM analysis allow the measurement of data like dry mass, sphericity, lipid content, protein content, and organelle identification [9]. HTM and AFM are complementary techniques since the first focuses on internal visualizing cell characteristics, whereas the second conducts high-resolution surface analysis.

Atomic force microscopy can be an accompanying technique to study cell morphology and mechanical properties [10]. Recent AFM microscopes have had significant modifications and advancements for their use in biological research [11]. Nowadays, AFM performs diverse analyses such as topographical imaging of cells, bacteria, and fungi, measurement of the mechanical and functional properties, recognition of molecules in organisms’ structures, manipulation of cells, and imaging of dynamic biological processes [2, 3]. Combining data generated by HTM (3D and internal view of cells) and AFM (high-resolution surface analysis, mechanical properties) is essential for developing fields like cell pathology, cancer research, microbiology, nanotoxicology, and many others**.** We pursue to optimize the synthesis variables to render diverse nanomaterials (NMs) with tuned properties for biomedical or environmental remediation applications [4–6]. In addition, numerous research efforts aim to implement standard protocols to evaluate NMs´ bio-activity since these materials might enter living organisms accidentally or deliberately [7]. In agreement with the previous statement, we prioritize gaining a complete understanding of the bio-activity of these NMs before their practical application. 4D high-resolution microscopy techniques (AFM and HTM) are flexible and robust strategies to study the intimal interaction of NMs (or any other substance) with living cells. This review briefly describes these techniques and recent reports on their application to investigate cancer, microbiology, and nanotoxicology.

## Holotomographic microscopy (HTM) principle

The principles of HTM are based on the developments achieved in quantitative phase microscopy, where multiple imaging techniques such as microscopy, holography, and light scattering are combined. A more detailed review of the physical principles of microscopy of the quantitative phase has already been carried out by Park et al. [10, 12]. In this way, this work will focus on the fundamental aspects of the equipment’s operation and its most recent applications in areas such as cancer research, microbiology, and nanotoxicology.

In the last years, optical microscopy surpassed the diffraction limit of light, resulting in super-resolution fluorescence microscopy. Because of the super-resolution of this technique, some authors call it nanoscopy. The use of fluorescence nanoscopy (FN) helped researchers discover the structural details of subdiffraction cellular architectures [13]. Furthermore, by multi-batch labeling, researchers can monitor the dynamic changes of cells and exogenous agents due to their interaction. Super-resolution imaging has become a non-invasive method for nanoparticle (NP) bio-distribution assessment with applications for understanding and optimizing nanomedicine performance [14, 15]. Nowadays, FN provides a graphic description of the complex interactions of nanomaterials (NMs) and cells, helping researchers with a more rational design of nanomaterials for biomedical applications. However, Confocal fluorescence microscopy has limitations for application in living cells. Within the protocol of this technique, there is the need to use fluorescent markers, which results in cell damage.

**Fig. 1:**
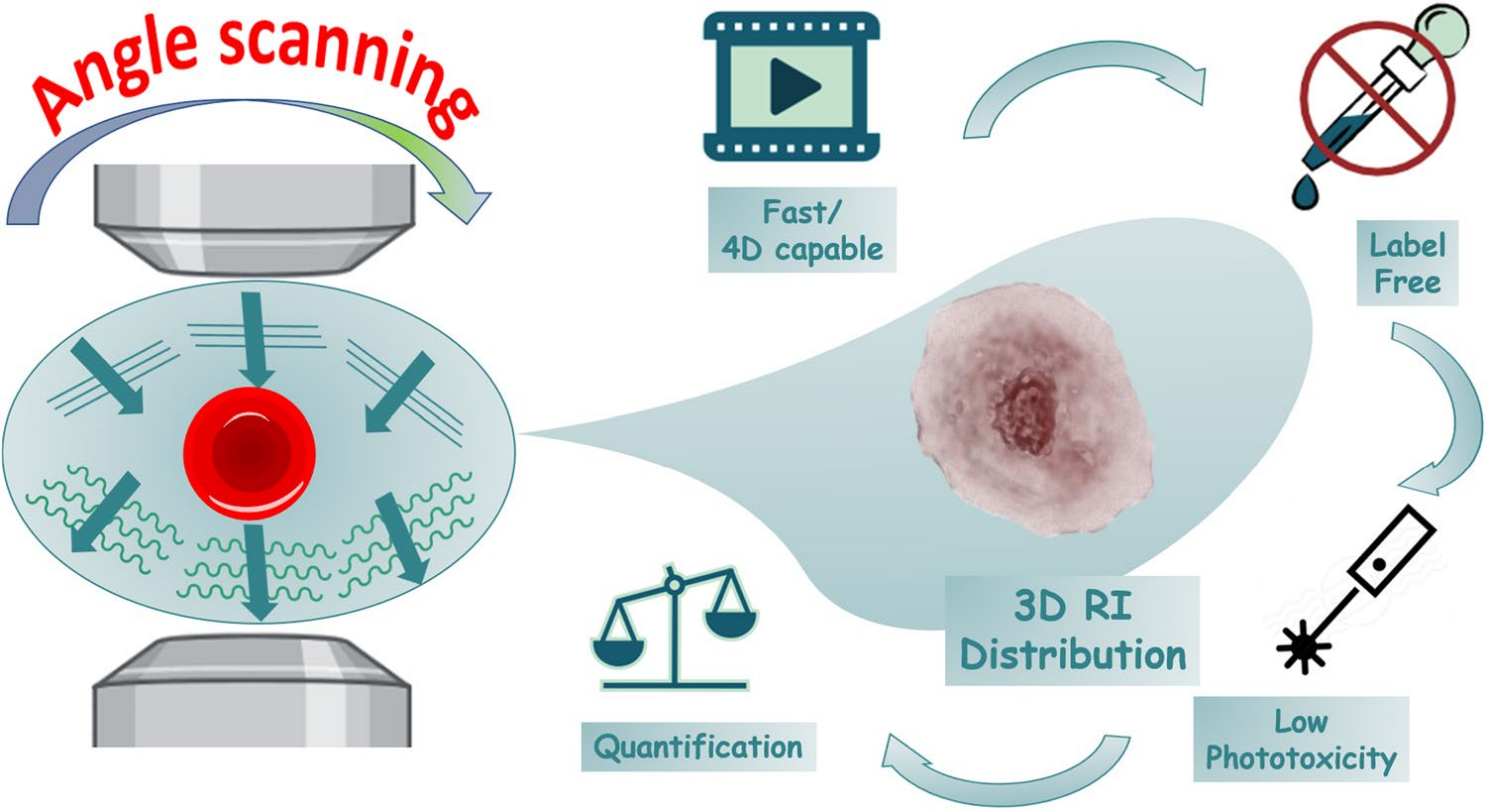
Main features of HTM. HTM is an emerging technique suitable for the fast 3D visualization of living cells and tissues. Imaging of living cells by HTM is conducted without using any preparation (fixation, staining). The technique also provides quantitative information (i.e., lipid content) of cells and organelles

Recently, HTM has emerged as a powerful label-free three-dimensional (3D) technique for imaging cell components without requiring cell staining and label-free nanoparticles (NPs) inside cells [16]. Modern HT microscopes offer advantages like no label requirements, low photo-toxicity, quick analysis, acquisition of quantitative data such as refractive index, dry mass, and protein content [17], which in most samples, the protein concentration is linearly proportional with RI values [18, 19]. HTM analysis permits the distinction of organelles without the necessity of fluorescent markers. Analysis of the expression of biomolecules present in organisms still requires labels if the organisms do not possess fluorescent nature. Figure 1 illustrates the main features of HTM.

Measuring the differences in the RI of cell components in living organisms to construct a 3D image of the cell is a complex task due to the light refraction in media with different refractive indexes, causing multiple light scattering effects. As a laser passes through a cell, the soft wavefront acquires distortion or phase information that can be converted into intensity [8], a feature that can be further measured and converted into qualitative data. For a 3D view of the cell, the microscope acquires multiple 2D holograms at various angles for image reconstruction [8]. HTM reconstructs the phase difference image of cells and provides the 3D RI and volume information of living and fixed cells. Because the RI value is proportional to molecular density is used for imaging cells (whole cells or intracellular organelles) and determining the molecular density of each organelle. For example, early applications of HTM included the classification of leukocytes, sperms, and red blood cells based in the differences on cell and organelle morphology (species-related properties) [9, 10]. It is also a valuable tool for investigating pathological characteristics of diverse cells [11], monitoring cell changes after exposure to different agents (chemicals, microorganisms, nanomaterials), and monitoring quantitative dynamics in cells and their organelles. For example, a recent study reports observing the morphological changes during apoptosis of C6 rat glial cells after exposure to methamphetamine hydrochloride [20]. Since HTM exhibits limitations on molecular-related information and the uncertainties of the structural reliability of organelles expressed only by RI, it is necessary to correlate HTM with complementary imaging techniques.

**Fig. 2.**
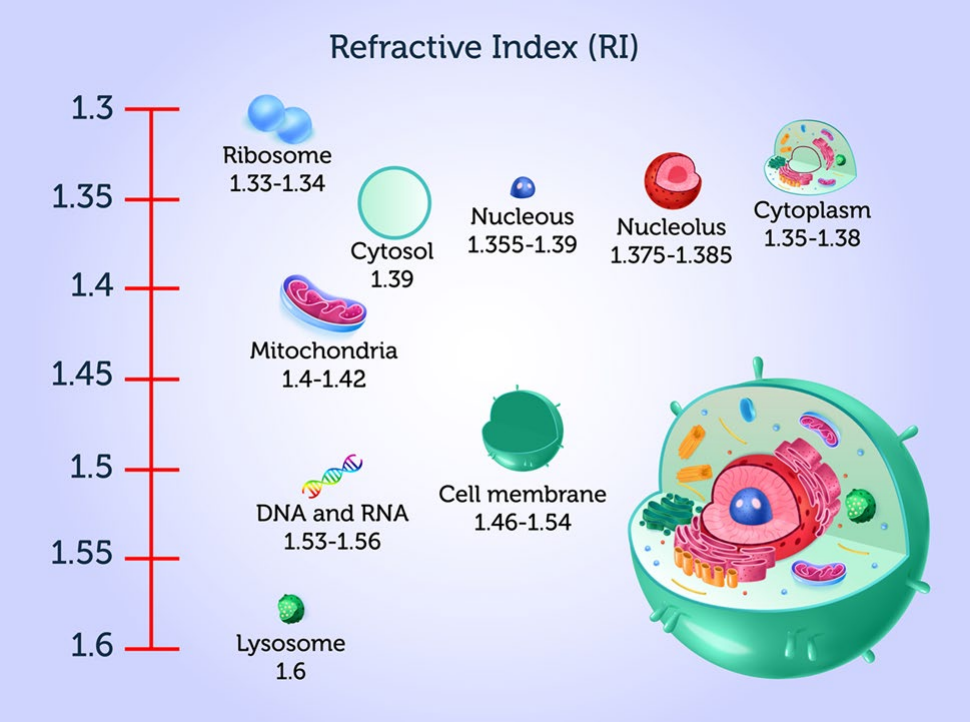
3D representation of a eukaryotic cell, with a schematic illustration of organelle refractive index. Cell organelles possess different refractive (RI) values. HTM relies upon this property (RI) for the 3D label-free imaging of living cells. As illustrated, some organelles have similar RI values, so care is needed for 3D image construction

As previously stated, many organelles possess different ranges of RI, permitting their identification by HTM. However, digital organelle staining requires selecting a range of RI so that knowing the refractive index values of various organelles is necessary for correct identification and digital coloring [12]. Table 1 presents a brief list of typical cell organelles and their RI values based on the literature. As can be noticed from Fig. 2, which includes the refractive indexes of cell organelles, many share RI values, so care must be taken when processing data to avoid confusing elements that can have similar RI values. HTM limitations rely upon low molecular specificity due to shared RI values among several proteins [8].

**Table 1:** Refractive index values of several cell organelles as reported in the literature

Organelle	RI value	References
Cell membrane	1.46–1.54	[13]
Cytoplasm	1.36–1.38	[14, 15]
Nuclei Nucleolus	1.375–1.385	[16]
Nucleus	1.355–1.39	[14, 16]
Extracelullalr fluids	1.35	[14]
Mitochondria	1.40–1.42	[14, 16]
Cytosol	1.360–1.390	[16]
Lysosome	1.6	[16]
DNA, RNA and ribonucleoprotein	1.530–1.560	[15]
Ribosome	1.330–1.340	[15]

**Fig. 3:**
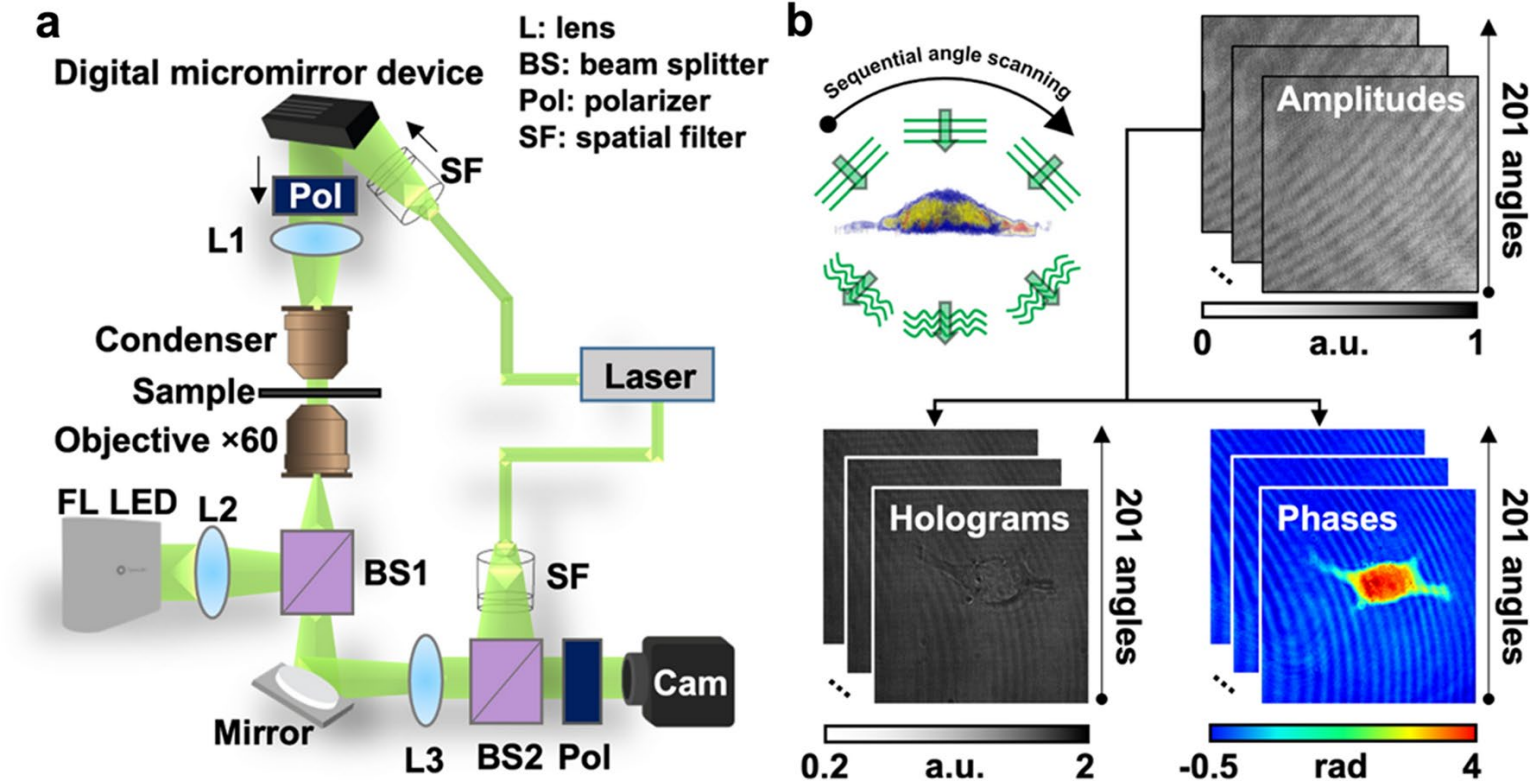
Holotomography microscopy principle. **a** Structure of an holotomographic microscope. The sample is situated between the objective and condenser lens and radiated with a laser beam. The laser beam splits to follow different paths (sample and reference). The sample and the reference beam combine to generate a 2D hologram. **b** Holotomography mechanisms for 3D image construction. The imaging laser beam radiates the sample with a specific incident angle, then the beam is rotated through 360 to the optical axis. From the overlapping taken holograms, the 3D (RI) tomogram of the sample is constructed [23]

Nowadays, HTM microscopes are commercially available. HTM microscopes differ in design, although modern equipment uses low energy wavelength for measurements, reducing its phototoxicity [17, 18]. Figure 3a illustrates a basic structure of an HTM microscope. A low-energy laser splits into two beams, one serves as a reference pattern, and the second interacts with the sample. The interference between the two beams is then used to construct a hologram [19]. The construction of a 3D hologram requires multiple analyses at different angles. Depending on the equipment, one can find different approaches to obtain multiple points of view in the samples (by using a moving mirror that deflects the laser in several spots to construct the image or by using a digital micromirror device) [21, 22]. Figure 3b shows a cell undergoing sequential angle scanning by HTM. HTM generates a refractive index (RI) distribution by putting together the RI range of images acquired from 201 sequential angles scanned from two-dimensional holograms [23].

As an emerging technique, HTM (also known as quantitative phase imaging, QPI) faces numerous challenges to becoming a routine technique for biomedical research. For example, there is the unavailability of a standard protocol to improve image acquisition [24], a lack of accuracy in 3D RI image reconstruction, limitations for imaging thick biological specimens [25, 26], and the need for standardized 3D QPI metrology. Researchers in the field; work on developing techniques to differentiate specific organelles in a cell and to distinguish single cells from neighboring cells. Modern HTM equipment includes software for 3D image processing; however, its algorithm can not discriminate precisely among the different cell organelles. Currently, researchers explore diverse strategies to provide a robust and general method to precisely and accurately segment cells during 3D reconstruction. Among these strategies are; computational models (machine learning techniques) [10, 27], the use of non-toxic RI matching media (iodixanol) [26], noise suppression techniques [28], and the standardization of image assessment metrics [29].

Despite the high specificity of fluorescence microscopy, the need for the use of fluorescent probes imposes several limitations for live-cell analysis. HTM lacks the molecular specificity of fluorescence microscopy but can distinguish different organelles or exogenous agents (NMs) because of their differences in RI values. A recent study investigates the efficiency of levofloxacin dry powder aerosols for tuberculosis treatment [30]. The authors used confocal laser scanning microscopy (CLSM) and HTM to look into the cellular uptake of the levofloxacin formulations. Both techniques show the internalization of the formulations by the NR8383 cells. In addition, HTM analysis demonstrates changes in the volume and concentration of the lipid droplets and the cell volume of treated cells. A different study reports the suitability of HTM to distinguish healthy from apoptotic cells and to monitor outer and inner cell changes [31]. Camptothecin-treated cells exhibit morphological (membrane blebbing and chromatin condensation) and biochemical (increase in lipid droplets) changes. Koutsogiannis et al. studied the effects of *Toxoplasma gondii* in host cells using HTM. They report changes in cell volume, dry mass, and surface area of infected cells [32]. Late HTM equipment integrates Artificial Intelligence to support automated single-cell segmentation and quantification [18].

**Fig. 4:**
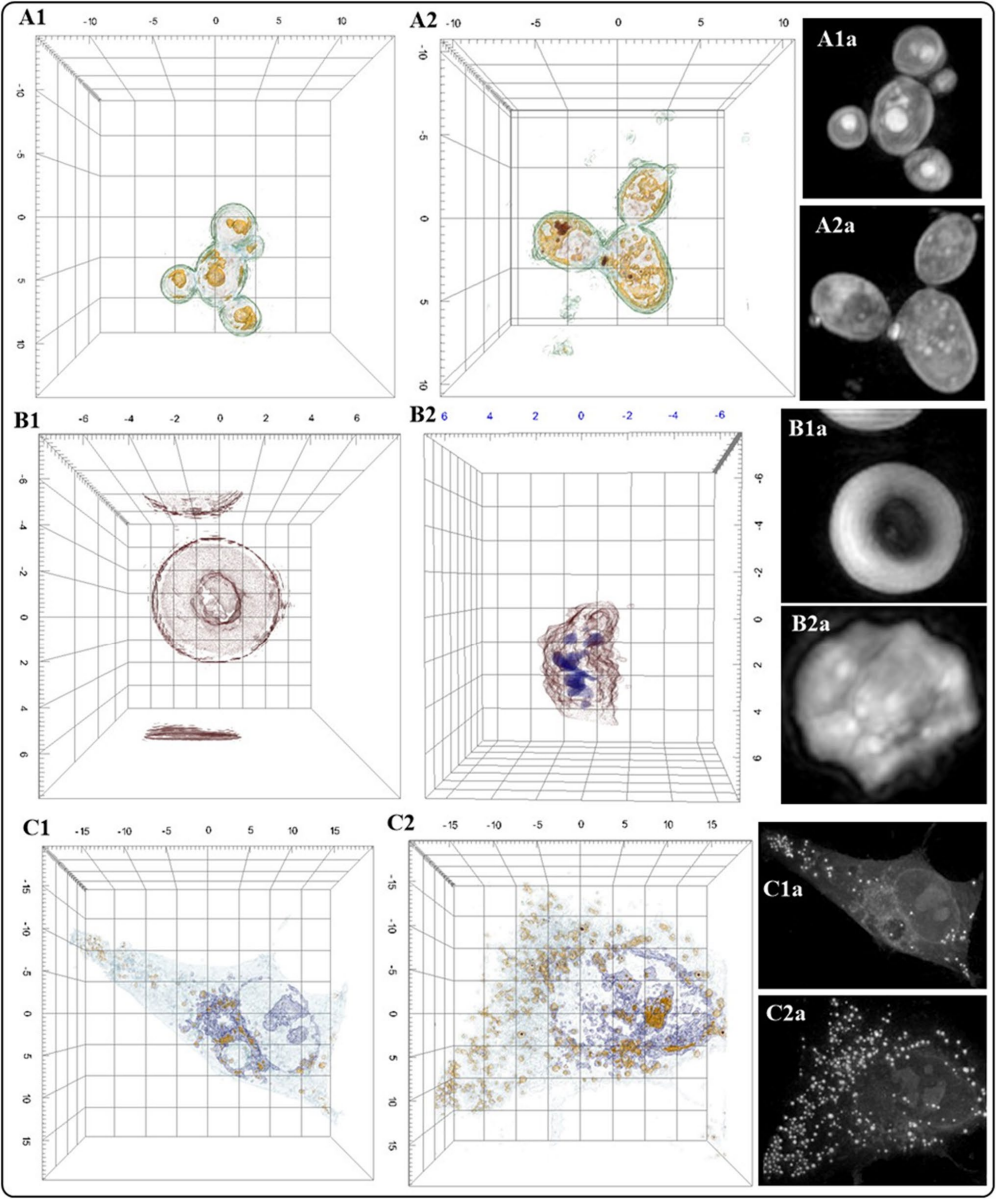
Examples of HTM images of different kinds of cells or organisms. **A1** 3D representation of *Candida parapsilosis* (*C. parapsilosis*)*;* **A2** 3D representation of *C. parapsilosis* exposed to NMs. NMs represented as red dots. **A1a** 2D representation of *C. parapsilosis* based on differences of refractive index; **A2a** 2D representation of *C. parapsilosis* exposed to NMs; **B1** 3D representation of a human erythrocyte; **B2** 3D lateral view of human erytrhocyte exposed to NMs. Blue dots represent NMs; **B1a** 2D representation of human erythrocyte based on differences of refractive index; **B2a** 2D representation of human erythrocyte exposed to NMs. Brigthest dots correspond to NMs; **C1** 3D representation of A549 cell; **C2** 3D representation of A549 cell exposed to NMs. NMs represented in red dots; **C1a** 2D representation of A549 cell based on differences of refractive index; **C2a** 2D representation of A549 cell exposed to NMs

Thanks to holotomography, many cells and organisms can be analyzed in great detail in record time due to its ease of preparation compared to techniques that need staining, which can take hours to prepare. Besides, the HT microscope can be operated under different conditions a characteristic that allows for the analysis of living cells (adherent or suspended), or living cells interacting with exogeneous agents (Fig. 4). Manipulation of the HT microscope is simpler than other electronic microscopes and its price is lower. HTM offers numerous advantages to advance live cell imaging: (a) high resolution (nanoscale), (b) label-free, (c) real-time, (d) quantitative phase imaging, (e) fluorescence. Figure 4 shows examples of HTM images of different kind of cells or organisms [4, 5]. Figure 4C1 shows a 3D representation of an A549 cell (control group). The cell shows unaltered size and morphology. Upon interaction of A549 cells with Au NMs, we observe changes in the morphology of the cell and an increase in lipid drop production (Fig. 4C2). Figure 4C1a and C2a are the 2D representation of the cells.

The versatility of HTM allows the combination of the microscope with other analytical techniques to increase the capabilities of this technique. A recent study by Ryu et al. [33], reports on combining a microfluidic device and HTM for red blood cell histopathological analysis. The microfluidic device was attached to the HT microscope to obtain biochemical (hemoglobin content) and morphological properties (corpuscular volume). It is also possible to couple the HT microscope with mass spectrometry for a more precise location and identification of organelles’ composition. Using live single-cell mass spectrometry coupled to HTM renders an improved 3D spatial resolution (X–Y-axis 0.18 µm and Z-axis 0.33 µm) and more accurate quantitative cell analysis [34]. As previously stated aquiring molecular specificity by HTM is still a challenge. A late study reports the construction of a computational mid-infrared photothermal microscope, which is able to obtain infrared spectra and bond-selective 3D refractive index maps [35].

As an emerging technique, HTM still faces challenges for reproducibility with data generated across research groups. To alleviate this problem, researchers must establish standard protocols for sample preparation and libraries with data regarding the RI values of organelles within different cell lines. Moreover, recent studies discuss that cell fixation can alter the refractive index of cells and cellular compartments, reducing the accuracy and reproducibility of HTM morphology analysis [36]. Despite the current practical limitations of the HTM technique, as outlined above, the growth ability of HTM depends on the user´s needs and technological advances. In this regard, the latest HT microscopes are suitable for analyzing organoids or tick specimens [37]. We want to remark that HTM is a versatile technique to image and analyze the dynamics of biological events because of its label-free and quantitative imaging capabilities. Among optical cell nanoscopy techniques, the advantages of HTM are its non-invasive nature, simplicity, and fast acquisition times. HTM renders images with high precision and high resolution (differences in the refractive index within the cell give the contrast for imaging different organelles). HTM provides a 4D examination of living cells under varying conditions (external stimuli or exogeneous agents –drugs, toxins, nanomaterials-) [38, 39].

## Atomic force microscopy principle and imaging modes

AFM has become one of the most versatile techniques for analyzing samples with many surface characteristics. Its main principle relies on deflecting a cantilever caused by the interacting forces between a tip attached to it and the sample. In most AFM, a laser is reflected on the cantilever surface to detect the cantilever deflections and know its position along the z-axis [40]. AFM has multiple image modes that adapt to different characteristics of the sample and allow obtaining diverse types of information based on the properties of the specimen and the AFM mode of operation [41]. In the beginning, AFM was not attractive to conduct research in the biological field due to the complex nature of the technique (setup, alignment, and adjustment of system parameters were cumbersome). Also, there were the limitations of the low Spatio-temporal resolution, mode of operation, and small scan area of the AFM types of equipment. The first commercial AFM microscopes operated under contact mode. Under these operation conditions, it becomes difficult the analysis of heterogeneous surfaces or soft samples [42]. Numerous technological advances allowed the implementation of different types of interaction of the AFM probe with the analytes, broadening the applications of this technique [43, 44]. The field of AFM has advanced rapidly in recent years with the integration of several modes of operation (AFM) with diverse optical, spectroscopy, artificial intelligence (AI), deep learning (DL), and, machine learning (ML) techniques [45–49].

### Application of AFM in biological research

AFM is a consolidated technique that allows a multiparametric analysis of living cells. Furthermore, AFM is the only system suitable for the label-free imaging of nanoscale biomolecular dynamics, which allows a deep understanding of molecular-scale mechanisms that cannot be investigated by other super-resolution imaging techniques. We refer the reader to previous reviews by [46, 50–54] that exhaustively describe the breakthrough discoveries that position AFM as a valuable tool for life sciences and biomedical research. Applications of AFM in the biomedical field increased with the development of tapping mode since the analysis of soft samples using intermitted contact (tapping mode) minimizes sample deformation. There are numerous examples of AFM application in the tapping mode (and its variances, HS-AFM, AFM-IR, AFM-SECM) to study live-biological specimens (microorganisms, tissues, or cells) [55–58]; or the interaction of exogenous agents (pharmaceuticals, toxicants, NMs) with living cells [59, 60].

**Fig. 5:**
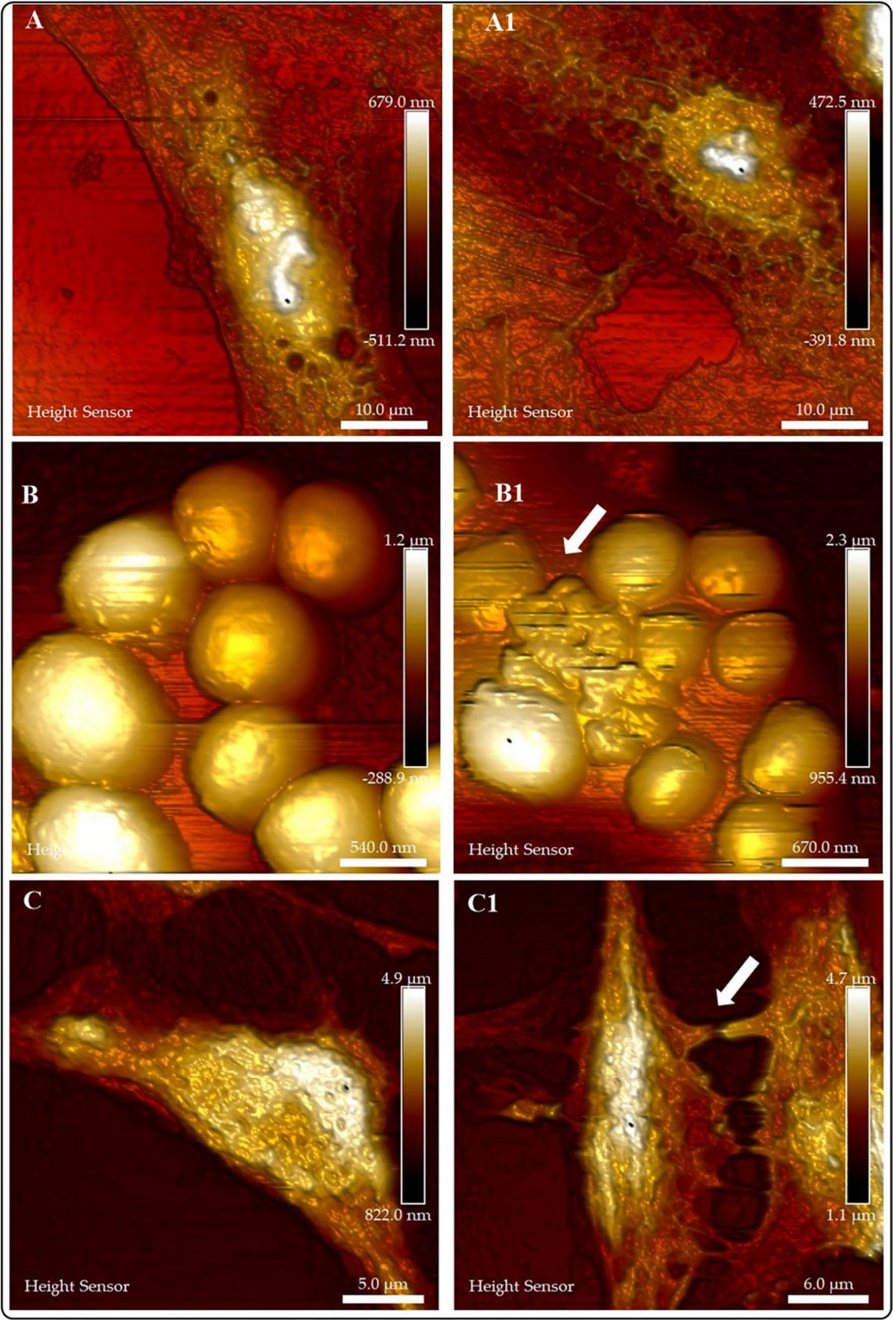
AFM analysis of the bio-interactions of NMs with cell lines and microorganisms. **A** A549 cell (control). **A1** A549 cell treated with Au NMs. B) *Staphylococcus Aureus* (Control). **B1** *Staphylococcus Aureus* treated with CuFe_2_O_4_ NMs. **C** SHSY-5Y cell (Control). **C1** SHSY-5Y cell treated with ZnO-Al^3+^ NMs

Our research group is interested in establishing AFM as a routine technique to study the bio-interactions of nanomaterials with different cell lines and microorganisms [6, 59, 61, 62]. Our results demonstrate the high resolution of AFM to elucidate the morphology and morphology changes of cells (or microorganisms) due to the interaction with nanomaterials. However, since AFM (traditional tapping, nowadays, nano endoscopy allows observation inside living cells) is a microscopy technique for high-resolution surface images, the AFM data does not demonstrate the entry of NMs in cells (or microorganisms). Figure 5 depicts AFM images of cells or microorganisms before and after interaction with nanomaterials (NMs). Figures 5A (A549 cells control group) and 5A1 (A549 cells treated with Au NMs) illustrate the changes in the morphology of the cells resulting from cell-NMs interaction. We previously discussed (Fig. 4C1 and C2) the morphological and biochemical changes (increase in lipid drop production) observed in these cells upon interaction with Au NMs. In contrast to HTM imaging, AFM renders a high-resolution image with morphological changes corresponding to HTM imaging.

We fabricate CuFe_2_O_4_ to investigate their antimicrobial activity under different stimuli (results to be submitted). Figure 5B and B1 show the damage exerted by CuFe_2_O_4_ NMs in *Staphylococcus Aureus*. White arrows indicate the presence of NMs in the bacterial cells. Figure 5C and C1 show the effects of the interaction of ZnO-Al^3+^ NMs and SHSY-5Y cells [4]. The control group (Fig. 5C) presents a typical morphology and connections with neighboring cells; on the contrary, treated cells (Fig. 5C1) exhibit changes in the morphology and damaged neighboring cell connections (white arrows).

**Fig. 6:**
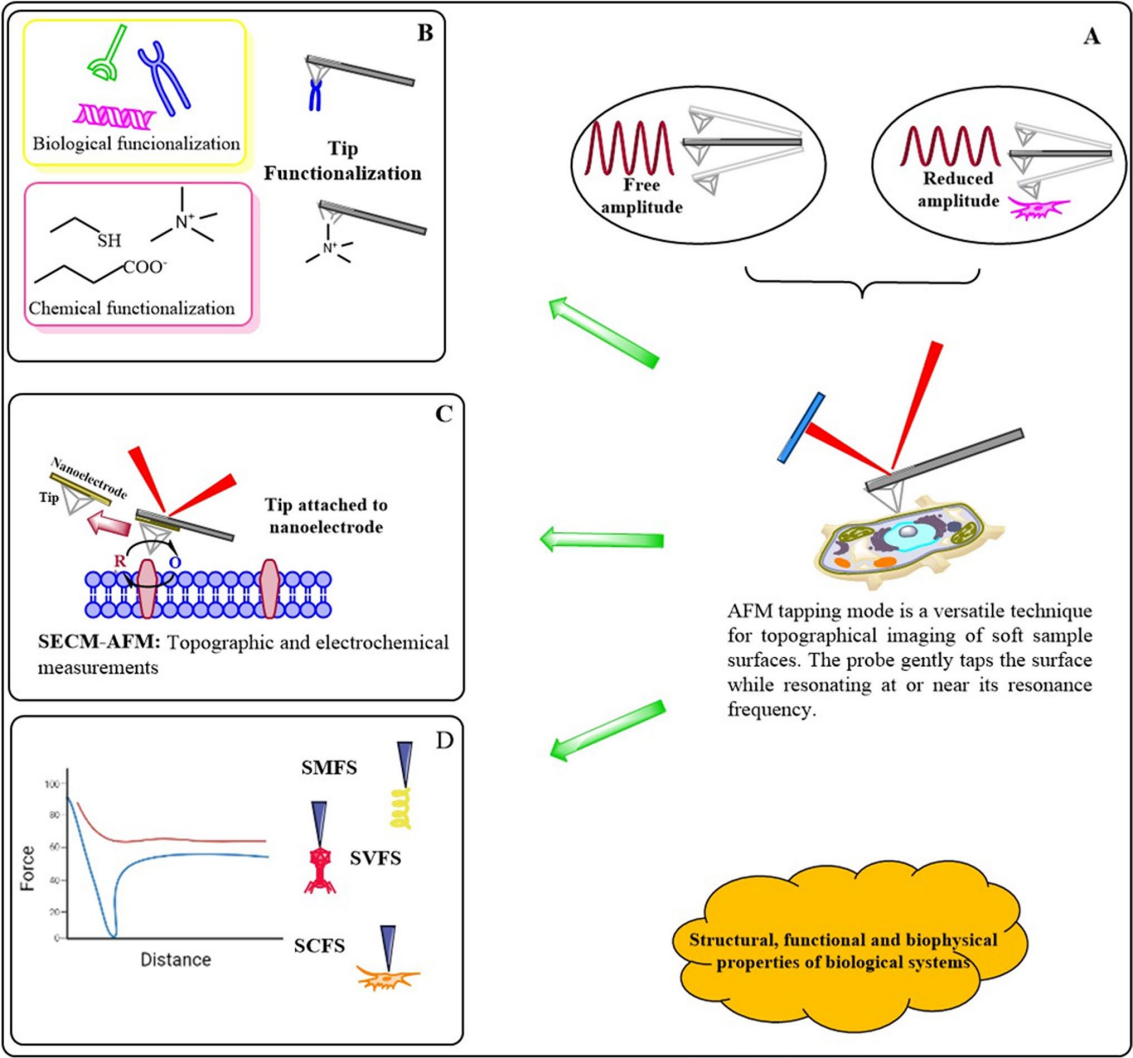
The use of AFM to examine (BIO)Chemical properties of biological systems (cells, cellular components, living tissues). **A** Dynamic Atomic Force Micorscopy is a suitable technique for biomedical research. **B** Biological functionalization of AFM tips can be used to examine topographic and biomechanical properties (binding –unnfolding proteins- and rupture –to pull apart single receptor-ligand complexes- forces). Chemically functionalized tips can be used to probe hydrophobic, hydrophilic domains or electrostatic interactions. **C**, **D** SFMS (Single molecule Force Spectroscopy) uses tips derivatized with biomolecules, drugs, viruses (SVFS; Single Virus Force Spectroscopy) or cells (SCFS; Single Cell Force Spectroscopy) to study ligand receptor interactions

With the advent of tapping mode, AFM became a dynamic tool to acquire the structural (micro or nanoscale) details and mechanical properties of cells or biomolecules (Fig. 6). Besides, technological discoveries allowed the implementation of tapping mode varieties (force nanoscopy, HS-AFM, and correlative microscopies). Numerous developments aid the complexity of AFM biological applications; for example, by modification of the AFM tip, it is possible to measure different surface properties, such as adhesion forces, friction, viscoelastic properties, mechanical properties (Young modulus) or electrostatic and magnetic properties (Fig. 6B). It is also possible to attach the tip to a microelectrode (conductive tips) to combine tapping mode with a different scanning probe microscopy (SPM) technique to study at the same time distinct surface properties (i.e., structural and electrochemical imaging) (Fig. 6C). Using this hybrid technique allows researchers to relate topography with conductivity maps, reveal the presence of specific molecules on the cell’s surface, or monitor its metabolic rate. The combination of scanning electrochemical microscopy (SECM) with AFM in tapping mode (SECM-AFM) allows the investigation of the activity of numerous molecules. Furthermore, AFM microscopy provides endless possibilities for tip functionalization (molecules, viruses, cells) to react selectively with a target molecule (receptor) to investigate specific biomolecular interactions.

Cells in living systems readily sense and respond to numerous and diverse mechanical signals; thus, nowadays, this response is investigated as an indicator of cell wellness or disease progression. For example, the mechanical stiffness of the surrounding extracellular matrix (ECM) critically determines normal cell function, stem cell differentiation, and tissue homeostasis. In contrast, abnormal changes in ECM stiffness contribute to the onset and progression of various diseases, such as cancer and fibrosis [63]. Accurate measurement of the mechanical properties of live cells is of utmost importance in today´s biomedical research. AFM is affordable to render a qualitative mechanical property mapping using intermittent mode (phase imaging). Furthermore, force spectroscopy and force volume allow the measurement of tip-sample forces at a point or over an area; regrettably, both techniques encompass long acquisition times. Modern AFM techniques offer multifunctional tools to measure the cell´s mechanical properties under physiological conditions or the application of *controlled* mechanical stimuli to evaluate the response of biological systems. The mechanical properties measured by AFM comprise force, pressure, tension, adhesion, friction, elasticity, and viscosity [64]. The combination of AFM with other complementary techniques allows the measurement of mechanical, functional, and morphological properties and the biological response of complex systems [65]. We refer the reader to recent reviews on AFM or correlative microscopy techniques to investigate mechanobiological properties [66].

Late reports remark on the recent discoveries that help AFM expand its reach. Penedo and co-workers discuss the implementation of Nanoendoscopy-AFM (3D-AFM), a new mode of AFM operation that allows the visualization of intracellular structures [67]. In this technique, inserting a needle-like nanoprobe into a living cell allows real-time (2D and 3D) imaging of intracellular nano dynamics without decreasing cell viability. This method surpasses the AFM limitation to 2D surface imaging, imaging unsupported 3D structures. The following studies comment on some technical considerations and applications (to observe organelles in living cells) for nano endoscopic imaging [68]. A recent review summarizes the main applications of 3D-AFM to study 3D self-organizing systems (3D-SOSs) with particular emphasis on intracellular components inside live cells [69].

### High-speed AFM (HS-AFM)

Despite the numerous advantages of AFM imaging, acquisition time was an obstacle to study biochemical or conformational changes of biomolecules in real-time. HS-AFM notably increases the image acquisition speed becoming suitable for studying biological processes in real-time and analyzing the dynamics of cellular processes [70]. One limitation of HS-AFM is the scanning area; however, during the last decade, the advances in electronics, cantilever and piezoelectric scanner design, allow to extend the available scan range (in air and in liquids). A recent study, remarks the importance on the innovations of modern AFM equipments, that operate at high speed, are stable in liquids, with large field of view and image with high spatial and temporal resolution. These characteristisc allow for imaging at high-speed under physiological environments to investigate and record the activity (dynamics) of different biomolecules (i.e., mithocondrial DNA replication) or live cells in real-time and at the single-molecule or single-cell level [71].

### Fluid AFM and FluidFM (fluidic force microscopy)

The possibility of AFM imaging in liquids allows the study of *living cells* under different conditions. The capability of AFM to *image* and *interact* with the surfaces of living cells or tissues under physiological conditions gives researchers endless possibilities to develop new methodologies for more realistic and accurate biomedical applications [72]. Initially, researchers used a liquid cell for AFM imaging in a confined physiological environment. Unfortunately, the volume of the liquid cell is an obstacle for many AFM biological applications since many biomolecules become diluted [73]. Later, by combining microfluidics technology and redesigning cantilever and cell liquid chambers, Fluid AFM imaging improved considerably.

The fabrication of hollow cantilevers, whose design is application-dependent, made possible the fluid force-controlled manipulation of single cells (Fluidic Force Microscopy -FluidFM-). Numerous biological processes can be investigated at the single-cell level using FluidFM [66]. For example, cell adhesion is essential for several functions of microbial (biofilm formation, survival, pathogenesis) and mammalian (embryonic development, tissue morphogenesis, inflammation) cells. FluidFM enables the quantification of living cell adhesion forces in a physiological environment. Recent reviews highlight the biological applications of the FluidFM technique [60, 74].

### AFM-IR

Traditional AFM cannot discriminate the materials composition of the sampled surface unless several properties like elasticity, electronic and magnetic properties are studied [75]. With the coupling of infrared spectroscopy, AFM opened doors for specific molecular analysis along the surface. When irradiated with light, the sample suffers thermal expansion that the tip can measure, relating the tip response with the excitation wavelength IR patterns can be obtained for acquiring chemical structural information [75].

AFM-IR nano spectroscopy is a sensitive technique that allows direct visualization of the drug loading of single biomolecule carriers, aiding in optimizing the protocol of biomolecule drug loading. Hanke et al. successfully applied AFM-IR to investigate the loading of DNA origami nanostructures with the photosensitizer methylene blue. AFM-IR finds application to study molecular changes in living bacteria.A recent review describes the application of AFM-IR for drug delivery systems (polymer-based, lipid-contained, and metal-based) characterization. For more information regarding the recent technological AFM-IR developmente, the reader is reffered to [45, 76].

### Correlative microscopy

AFM is nowadays a robust and multifunctional technique for manipulating and detecting bio-interactions at nanometer resolution. However, as previously discussed (AFM-IR), AFM cannot provide information regarding chemical composition or internal structures. To solve these limitations, AFM can be combined with other microscopy techniques. For example, super-resolution optical microscopy can simultaneously identify several cell components (or exogenous agents) and visualize inside the cell. Advanced AFM equipment for life sciences combines optical and AMF techniques to avoid the inherent limitations of single microscopy techniques. Utilizing correlative data facilitates understanding the complex relationship between structure, composition, and function by picturing functional information in the contact of structural and compositional details in biological research [52]. For more information on the advances in correlative AFM and optical microscopy, we refer the reader to [77–79].

Different AFM types of equipment exist nowadays commercially. Of utmost importance is that these pieces of equipment are user-friendly since they can operate under automated conditions (setup, alignment, and re-adjustment of system parameters). Furthermore, these microscopes offer a high Spatio-temporal resolution, large scan area, and fast scanning of corrugated samples becoming, very attractive for biomedical applications. AFM is not just an imaging technique; it also allows the determination of the analytes’ different nano-mechanical properties (stiffness, elasticity, dissipation, viscoelastic properties, hardness, among others). Latest generation microscopes can also operate under numerous conditions (temperature, air, in fluids). Data analysis is simple, accurate, and versatile (it is even possible to overlay AFM data with data generated with another advanced optical microscopy). For example, the combination of confocal microscopy and AFM indentation allowed the study of cell mechanics in 3D environments, demonstrating the change in the mechanical properties of metastatic cancer cells during invasion into collagen I matrices [80].

## Atomic force and holotomography microscopy in cancer, microbiology, nanotoxicology and nanomedicine research

### Cancer research

Microscopy techniques are versatile strategies that contribute to widening our understanding of molecular processes, including the biology of numerous diseases [78]. For example, cancer biology is very complex, and it is necessary to use different approaches to comprehend its growth mechanism and offer solutions to cure this disease. Microscopy can efficiently complement the data generated by conventional techniques (immunoblotting, PCR, flow cytometry) to study and diagnose cancer. In particular, AFM and HTM allow the visualization of living cancer cells and the changes they undergo during their growth or while interacting with exogenous agents. One of the main advantages of using AFM and HTM for biomedical studies is the no-labeling analysis, avoiding damage from the dye (composition or upon excitation). In the last decade, AFM has become a flexible tool for studying the biophysical properties of native and modified cells. On the other hand, HTM (as an emerging technique) faithfully reproduces the 3D morphology of living cells and portrays the morphological changes of cells interacting with an exogenous agent (drug, pathogen, nanomaterial). These microscopy techniques offer numerous opportunities to widen the type of In vitro studies that can bring more opportunities to find solutions to cure infectious or chronic diseases (i.e., cancer).

Cancer is the second leading cause of death in the world. Despite the research efforts to find efficient treatments to cure this disease, the current cancer treatment methods are not selective cancer therapies, resulting in adverse effects in no target organs [81]. Furthermore, present diagnostic techniques for metastatic cancer cells are invasive and complex (identification of metastatic cells in lymph nodes can be difficult, increasing the probability of inaccurate results); this fact highlights the need for alternative and complementary strategies to support physicians with a metastatic cancer diagnosis since an early cancer diagnosis favor appropriate treatment and patient survival.

AFM is useful for studying the mechanics of cells to differentiate cell physiological and pathological states. It can also perform a high-resolution analysis of the ultrastructure of cells remarking differences between cancer and normal cells. For example, white blood cells from leukemia patients have a cell roughness higher than healthy white blood cells because they present needle-like structures on their surface. In general, cancerous cells experience physical changes in elasticity and adhesion [82]. In addition, Force spectroscopy AFM examination gives information on cells’ hardness, adhesivity, and Young modulus. The knowledge of these properties aid researchers in determining if the cells are cancerous, invasive, or metastasic. They also help to elucidate the effects of drugs on cancer cells [83].

AFM can become a powerful strategy for early cancer diagnosis. For instance, AFM can point out differences between single cells, helping to identify cancerous and non-cancerous cells at early disease development. Besides, for some types of cancer (brain cancer, for example), tissue biopsies are risky for the patient. Liquid biopsies are an alternative for the early diagnosis of these pathologies. However, the analysis of these biofluids by traditional techniques can be cumbersome. The composition of the biofluids is heterogeneous, but some of its components can be cancer-specific material, and their identification contributes to early cancer diagnosis. In biofluids, exosomes are important cancer biomarkers. These molecules participate in cancer advancement and metastasis by transferring bioactive molecules between cancerous and non-cancerous cells. Using AFM (nanoindentation) is possible to determine the mechanical properties of these molecules and correlate them with the cancer stage [84].

AFM also finds applications in determining the effectiveness of cancer drugs. The study of the mechanical properties of cells found application in identifying cancerous cells since cancer cells (thyroid, ovarian, breast, prostate, bladder, pancreas) have a lower Young’s modulus than healthy cells. This knowledge now applies to investigating the efficacy of cancer drugs. For example, a recent review summarizes the use of AFM to examine the efficiency of cancer drugs directed to the cancer cell cytoskeleton [85]. Another study describes using a microfluidic device to study the interaction of living cancer cells (A549, MDA-MB-231, and MDA-MB-231/BRMS1) with doxorubicin; monitoring the effects of the drug by Raman spectroscopy and AFM [86]. Cancer multidrug resistance is a biomedical challenge. Numerous research efforts seek a better understanding of the molecular mechanisms that result in cancer multidrug resistance, then apply this knowledge to develop solutions to this medical problem. AFM and single live-cell imaging are suitable for identifying multidrug resistance phenotypes in cancer biological specimens. They are also appropriate to monitor the fate and behavior of an individual cancerous cell in complex cancer models [87].

Holotomography microscopy is also a versatile tool to study the conformational changes of cancer cells, their composition, or the effect (chemical or morphological changes) of anti-cancer drugs. Because of the emerging nature of this technique, the number of reports using HTM to study cancer cells is smaller than in AFM studies. However, some literature reports demonstrate the feasibility of using HTM to aid cancer research. For example, recent research uses HTM to monitor morphological changes in cancerous cells that received photodynamic therapy (PDT). They evaluate the effect of curcumin in cancerous cells at different doses and under radiation. Curcumin acts efficiently as a photosensitizer to inhibit the growth of melanotic melanoma cells. After treatment, they evaluate the viability and conformational changes of treated cells. PDT using curcumin as a sensitizer induce cytoskeleton reorganization in melanotic melanoma cells (A375) [88]. HTM is also helpful in evaluating the efficacy of electrochemotherapy and the influence of 17ꞵ-estradiol on the treatment of ovarian cancer. Assessment of morphological changes in MDAH-2774 cells by HT microscopy, contributes to determining treatment efficacy [89].

HTM is advantageous in supporting automated cancer screening. Overall, cancerous cells have a higher average RI than their healthy counterparts. They also have morphological changes like increased nuclear size, irregular shape (cellular and nuclear), and increased chromatin content. Furthermore, monitoring of apoptotic cell death is possible by HTM. Evaluation of apoptotic changes in suspended and adherent culture cells is possible using HTM. Several examples show that HT microscopy accomplishes detecting structural changes in cells and thin tissues undergoing apoptosis. In particular, a recent study shows a comparison of the results of different microscopy techniques (SEM, TEM, and HTM) to image the effects of camptothecin in U937 (human myelomonocytic lymphoma cell line) [31]. Although the morphological analysis is similar, the simplicity and speed of image acquisition of HTM are advantageous over the other techniques.

Intensive research is undergoing for the early detection of metastatic cells. A recent investigation searches for morphological and molecular differences between isogenic cell lines (P231, CTC, and LM) with diverse metastatic potential using HTM and Raman spectroscopy. HTM analysis acknowledges the morphological and molecular differences among cell lines. Raman maps assist in analyzing the metastatic potential of these cells [90]. Another research uses a combination of imaging phase microscope and machine learning strategies for cell analysis and classification during cell flow (cells flowing in a microfluidic channel). This approach is adequate for identifying circulating tumor cells of colorectal adenocarcinoma in liquid biopsies [91].

Recent reports hallmark the applicability of HTM for the study of the efficiency of cancer drugs. For example, D’Brant et al. demonstrate that glial cells suffer apoptosis upon methamphetamine treatment. Palacios-Acevedo et al. [92] studied the effect of clopidogrel as an adjuvant treatment that avoids cancer-associated thrombosis and tumor growth in pancreatic cancers. They use HT microscopy to investigate morphological and dry mass changes in pancreatic cancer cells treated with ticagrelor. HTM is also appropriate to visualize the effect of ablation techniques (irreversible electroporation) in cancer cells [93]. Another study describes the apoptosis of HeLa cells mediated by AS-DK143-loaded mPEG-PL NPs. HT microscopy shows the formation of apoptotic bodies and lipid accumulation that occurs in a time-dependent manner [94].

Of utmost importance is finding solutions to cancer multi-resistance and cancer relapse. The determination of cancer-drug resistance using traditional approaches is expensive and time-consuming. Thus, there is a need to create new strategies to evaluate cancer-drug sensitivity. Novel approaches can help to solve this problem. Confocal fluorescence microscopy is a suitable strategy to assess the drug resistance of cancer cells. Unfortunately, for some cancer types (epithelial ovarian cancer, EOC, for example), the clinical protein markers still do not exist. To overcome this problem, investigating changes in the morphology of cancer cells helps to determine cancer drug sensitivity [95]. For instance [96], shows how to assess cancer-drug resistance in epithelial ovarian cancer (EOC) cells using a microfluidic flow cytometer adapted to an HT microscope strengthened by machine learning. A different study deciphers the biological mechanisms of simultaneous resistance to several chemically diverse cancer drugs. HT microscopy effectively identifies cancer markers responsible for multi-resistance [97].

Latest studies reveal that advanced microscopy techniques contribute to understanding the behavior of cancer cells and their interactions with therapeutic agents [82]. They also apply to study multidrug resistance of cancer cells avoiding inappropriate treatment of patients [87]. HT and AFM microscopies are inovative tools that can be used for *non-invasive* simultaneous monitoring of morphological, mechanical and chemical changes in cells during disease development (i.e., apoptosis) and can also be used to monitor other dynamic cell processes.

Biological systems and their processes are very complex becoming necessary to use several tools to investigate their natural and non-natural (pathological, toxic exposure, drug treatment) states [98]. Microscopy is a powerful strategy that permits scientists to visualize cells, tissues, and molecular processes in real time. The use of complementary and synergic imaging strategies provides a powerful combination of specificity and detailed structural information [87]. Accordingly, correlative microscopy finds numerous applications in deciphering complex disease-associated mechanisms [52]. For example, physicians experience a big challenge in cancer treatment due to the complexity of cancer biology. In this contribution we contend that we can improve our interpretation of the biological system under study by combining AFM and optical live-cell imaging (HTM). AFM can monitor in real-time structural (cells, single molecules, molecular complexes) and mechanical properties in cancer cells upon treatment with antineoplastic agents. In addition, HTM can perform fast imaging and 3D time-lapse response analysis of cancer cells (un-altered or upon antineoplastic application). It is also possible to evaluate the effects of drug treatment expressed as changes in the composition of cancer cells.

### Microbiology

There is an intense battle to find efficient solutions to the cure of infectious diseases. Resistance to antimicrobials is a serious concern since the number of resistant species and multidrug resistance increases. Recent advances in microscopy techniques can contribute to the research on the discovery of new antimicrobial agents. For example, AFM analysis reveals morphological and mechanical changes in microorganisms (MOs) after exposure to antimicrobial agents [57].

AFM finds numerous applications in biomedical studies since this technique offers both nanoscale imaging and nanomechanical characterization of biological materials in physiological environments. Late studies use AFM to investigate the effect of antibiotics on the mechanical properties of bacteria. For example [99], observed that only the virulent strain of *Bordetella pertussis* undergoes a decrease in height and elasticity upon antimicrobial exposure. Another study investigated the effects of different antibiotics (nitrofurantoin, furazolidone, and nitrofurazone) in probiotic bacteria, observing changes in bacterial cell morphology, topography, and adhesion parameters, changes that might impair biofilm formation [100]. A recent review highlights the use of AFM to understand the mechanism of antimicrobial drugs at the nanoscale level on microbial interfaces. The study includes an investigation into the alterations of bacterial cell morphology, nanomechanical properties, and adhesive abilities of microbes. Finally, it shows the use of AFM as NanoMechanicalSensors (NEMS) to detect quickly and accurately microbial resistance [57].

HS-AFM can measure structural changes in time frames of milliseconds, allowing real-time studies of the interaction of MOs and drugs. Furthermore, by using AFM, it is possible to study the interactions of bacteria with surfaces (evaluation of the adhesion properties at the molecular level) for the design of anti-adhesive therapies [54, 101]. A recent review highlights the applications of AFM in cellular and molecular microbiology [72].

Recently, hydroxyapatite has found a wide range of applications in medicine; mainly to regenerate bone or to fix bone defects in orthopedic, maxillofacial, and dentistry procedures. It is also suitable to coat prostheses to improve their biological properties [102, 103]. A worrisome problem with implant materials is the development of post-surgical infections. Recent studies demonstrate that Ag-hydroxyapatite nanocomposites prevent post-surgical infections. Stanic and collaborators investigated the antimicrobial activity of Ag-Hydroxyapatite against microorganisms responsible for implant-related biofilms. The AFM analysis shows the morphological changes of the fungal or bacterial cells due to Ag-Hydroxyapatite activity [104]. A more recent study investigates the nano-interactions of CuI-TiO_2_ NMs and fungal cells [5]. As with previous reports, AFM gives a high-resolution image illustrating the morphological changes of the fungal cells exposed to nanomaterials. However, it is not precise to conclude about the entry of the NMs in the fungal cells; thus, the use of HTM permits visualization of the NMs inside the cells, demonstrating the complementarity of these microscopy techniques.

The study of non-pathogenic MOs is environmentally relevant. Cyanobacteria and microalgae are important primary organisms in aquatic environments. A recent AFM (force-distance curves) study reveals the mechanical heterogeneity of the external layers of these MOs [105]. This study gives a better understanding of the biophysical mechanism that helps these MOs to adapt to turbulency. In another investigation, red algae, *Porphyridium cruentum* was subjected to nitrogen starvation and analyzed using AFM (tapping) to investigate if photosynthetic membranes are prone to alterations. As a result of nitrogen starvation, the photosynthetic membrane exhibit morphological and structural changes: (a) a slight increase in thylakoid vesicles, (b) the density and size of the phycobilisomes decreased gradually with time, (c) the presence of holes in the thylakoid membranes, possibly due to the loss of phycobilisomes, membrane rupture, or sample preparation [106].

High-speed AFM has been of great help in elucidating the dynamic phenomena of biomolecules and structures present on the surface or below the cell membrane. For example, Kobayashi et al. investigated the sliding mechanisms in the mycoplasma mobile parasite. The results obtained by HS-AFM showed spatial and temporal characteristics of the groups of particles present on the cell surface, showing particles with movements of up to 9 nm in times of 330 ms; these movements derived from ATP hydrolysis reactions result in cell movement [107]. In a different research, the HS-AFM (phase imaging) examination of nanometer-scale extracellular membrane vesicles (MVs) shows the differences in the properties of MVs among bacterial species [108]. On the other hand, the use of AFM to study microalgae has been increasing. However, it only represents about 0.17% of the research related to the study of microalgae; just like bacteria, studies on this type of microorganisms have focused on obtaining morphological characteristics, topography, mechanical and adhesion properties. Nevertheless, it has also been possible to study substances produced by microalgae, such as exopolysaccharides. Techniques such as Fluid-AFM can evaluate parameters such as lipid profiles to make the cultivation processes more efficient [109].

Some bacteria possess pili in their cells envelopes. These pili have numerous biological functions: adhesion, gene transfer, virulence and biofilm formation. Pili are also responsible of bacterial motion or endothelial cell invasion. A recent report describes on using AFM-based force-clamp spectroscopy to study pilus motility in *Caulobacter crescentus.* In this AFM modality, force is applied and kept at a constant level by continuosly adapting the position of the piezoelectric device. The results of this research demonstrate that force-clamp AFM is a novel tool to monitor pilus retraction. This knowledge can be applied to the treatment or prevention of bacterial infections [110]. AFM is also a powerful tool to study the interaction of MOs and host cells [111, 112]. By using single-molecule AFM analysis [112] demostrates the affinity of *S. aureus* (adhesins) to endothelial cell integrins under different conditions (Low or high stress; 100 pN or 1000–2000 pN). *S aureus* uses these proteins to invade epithelial and endothelial cells. They observe that structural changes occur under high stress favoring the integrins affinity to bacterial adhesins. Knowing the conditions that favor MOs infection facilitate the implementation of strategies to fight staphyloccocal infections.

Several microbial-related cases can benefit from holotomography studies (or studies combining HTs with other techniques). In recent research, they addressed the problem regarding the lack of facile and fast procedures for microbial identification. Modern technologies like mass spectroscopy are expensive and time-consuming. To solve this, the combination of holotomography and machine learning can lead to a method for the rapid identification of pathogens and diagnosis of patients with infectious diseases [113]. As previously discussed for AFM, HTM is also a potent tool to measure alteration in bacteria upon antibiotic treatment. Recent research shows HTM studies of the *in-situ* interaction of *Escherichia coli* and *Bacillus subtilis* to different concentrations of ampicillin. Their results include morphological (3D) and biochemical alterations of bacterial cells, which are ampicillin dose-dependent. They can also evaluate bacterial cell lysis due to changes in cell volume or cellular dry mass [114].

**Fig. 7:**
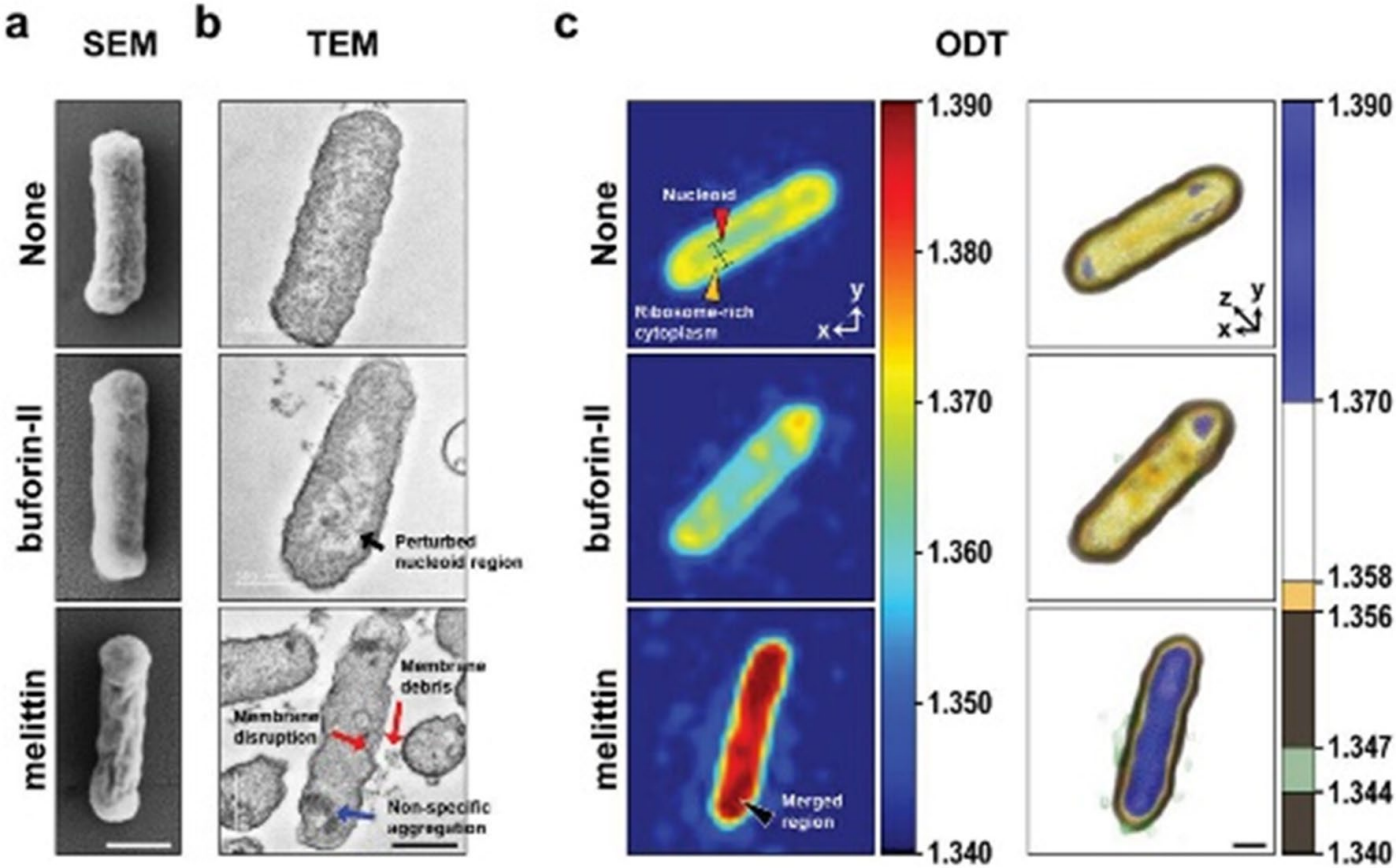
Comparison of Electronic Microscopy (SEM, TEM) imaging of *E coli* with HTM [115]

Kim et al. investigated the antimicrobial efficiency and mechanism of action of antimicrobial peptides (AMPs) using HT for Real-Time monitoring (Fig. 7). The fast and high-resolution imaging of HTM allows the investigation of real-time interactions between AMPs and bacteria. Their results clearly illustrate the antimicrobial mechanism (membrane disruptive compound) of AMP and the morphological alterations of bacterial cells [115]. As previously mentioned, the versatility of HTM allows its combination with other techniques to improve its applications. For example, combining HTM and deep learning results in the prompt optical screening of anthrax spores. The fast detection of anthrax spores demonstrates the suitability of HTM as a sensor in realistic settings of biological warfare [116].

Although more HTM studies focus on bacteria or fungi, parasites (such as protozoans) are also suitable for HT microscopy identification. Larrazabal et al. investigated the antiparasitic effect of ezetimibe in human and veterinary parasites (*Toxoplasma gondii*, *Neospora caninum*, and *Besnoitia besnoiti*). These parasites have many consequences in humans and animals, provoking several diseases. During asexual reproduction, they affect host cells. Through HTM, they observe that ezetimibe led to reduced meront sizes in the three parasites with no alteration of morphology of non-infected cells [117]. It is important to remark that HT microscopy can overcome the limitations present in other microscopy techniques. For example, investigations on sporogony are scarce in literature since the commonly used fluorescent dyes do not penetrate resistant oocyst bi-layered walls. HT microscopy is appropriate to study sporogonial oocysts and their alterations (time-dependent or anti-coccidial drug-derived effects) [118].

The versatility of HT microscopy and its suitability to study living cells allows the study of parasites’ life cycles. For example, by using 3D HT microscopy, it was possible the analysis of differing sporozoite egress [119]. Another study demonstrates the application of HT microscopy to conduct pathophysiological research. HT microscopy is appropriate to discriminate morphological and biochemical changes in the parasite (tachyzoite) and its infected cells [120]. A different study demonstrates the application of HT microscopy to differentiate the alterations in the host cellular cell cycle (bovine umbilical vein endothelial cells (BUVEC)) induced by different parasites (*Besnoitia besnoiti* and *Toxoplasma gondii*) [121]. HTM has recently been employed to investigate the *Toxoplasma* lytic cycle and to demonstrate alterations in volume, surface area and dry mass of genetically enginereed *Toxoplasma* mutant cells [32].

The application of HTM to study the interaction of pathogenic MOs with cells or exogenous agents can be numerous and depends on the user´s needs. For example, by using HTM, it was possible to observe the reaction of polymorphonuclear neutrophils to *Besnoitia besnoiti* bradyzoites infection [122]. A previous study from the same research group reports an increase in the production of lipid droplets (LDs) in *Besnoitia besnoiti* infected BUVEC cells [123]. Recent studies report the involvement of LDs in the cellular stress response (detoxification events or responses to different diseases in several cell lines) [124]. LDs can serve as bio-indicators of cell stress since they are dynamic organelles that regulate lipid uptake, metabolism, trafficking, and signaling in the cell. HTM can easily visualize and quantify LDs due to its elevated refractive index.

A recent study points out the unique advantage of HTM for the real-time monitoring of bacterial cells (morphological and biochemical changes) in contact with antimicrobial films. The authors discuss that several of the current methods available to investigate the antibacterial activity of 2D NMs (colony counting method, SEM, live/dead fluorescent staining) are limited in tracing the changes of the bacterial cells in contact with the NMs over time and observing continuous changes in bacterial membrane to decipher the antibacterial mechanism of 2D NMs [125]. Another study reveals the suitability of HTM to observe the virus-induced cytopathic effects in live cells. Their results demonstrate differences in the refractive index gradient of infected cells [126]. Furthermore [127], demonstrates the relevance of using HTM to investigate COVID-19 disease severity due to the presence of microclots even after the recovery period.

One of the first views of phytoplankton through 3D holotomography was achieved by Lee et al. (2014), demonstrating the capabilities of this technology for distinguishing organisms and obtaining parameters such as dry mass, volume, and cytoplasmic density [128]. Today, microscopy analysis of algae and phytoplankton can render distinction of cell parts, frustules, protoplasm, vacuoles, and chloroplasts using their RI [129], as well as the quantitative study of compounds of interest such as lipids present in microalgae for their use. in the production of biofuels [130].

### Nanotoxicology and nanomedicine

Nanotoxicology and nanomedicine are emerging disciplines; the first is responsible for evaluating the toxic effects of materials whose sizes (at least one dimension) do not exceed 100 nm [131]; the second seeks innovative solutions in the biomedical field by using nanomaterials (NMs). Numerous research efforts aim to implement standard protocols to evaluate NMs´ bio-activity since these materials might enter living organisms accidentally or deliberately. Nanotoxicology seeks to provide protocols and model materials for the systematic evaluation of the adverse effects of nanomaterials on cells, organs, or various more complex organisms such as plants or animals [132]. Nanomaterials possess unique physicochemical properties that differentiate their toxicological activities from their bulk counterparts [133]. Morphology and ion leaching are considered inherent properties of nanomaterials that affect their toxicity [131, 134]. Modern microscopy techniques are versatile tools that can aid to elucidate the mechanism of interaction of NMs with living organisms (from entry to the final destination).

Traditional toxicological assays are not always suitable to evaluate NMs toxicity due to the inherent properties of the NMs, composition of the exposure medium, or the response of the living organisms to NMs. For example, ion leaching from NMs can interfere with colorimetric assays, whereas protein corona formation might mask the biological activity of NMs [135, 136]. Lately, molecular spectroscopy and microscopy techniques have become potent tools to study the physical and molecular interactions of NMs and cells, offering alternatives for understanding NMs activity (or toxicity). These techniques can measure morphological changes and observation of the spatial disposition of the materials inside and outside the cells [137, 138].

As mentioned before, nanomaterials tend to interact with multiple components in a system depending on their physicochemical characteristics. Protein corona forms in protein-rich media, which gives new surface characteristics to NMs. A study reports the measurement of protein corona through AFM in graphene nanomaterials exposed to proteins present in commonly used culture media such as DMEM. The AFM (topographical) analysis of graphene shows significant accumulations of fetal bovine serum proteins along the surface of the nanomaterial. Protein adhesion increases the roughness and thickness of the graphene sheets. These changes in the material characteristics imply modifications in their toxicological behavior [139]. In a similar study, TiO_2_ and ZnO NPs were subjected to food matrices (sucrose, protein powder, and corn oil) to evaluate their interaction with the media. AFM revealed that surface adhesion was decreased in both particles when interacting with proteins due to the protein corona formation. On the other hand, corn oil caused an increase in surface adhesion related to high oil viscosity due to the long carbon chains [140].

To better address the challenges of nanotoxicology assessment, it is necessary to use multiple biological models (from simple to complex organisms) to avoid misleading results regarding the entry, transformation, and final destination of NMs in living beings. The use of eukaryotic cells or bacteria as model organisms is widely explored due to their simplicity; however, more complex organisms such as nematodes are necessary. NMs can alter the structure of the nematode cuticle, affecting its movement and protection from the environment. AFM is a versatile strategy to evaluate cuticle integrity (morphology and mechanical properties) after exposition to NMs [141]. For a more detailed toxicity mechanism of NMs, In vitro and In vivo studies should be performed. For example, on eukaryotic models such as HEK293T embryonic kidney cells, results showed that nanoparticle toxicity could be evaluated through biomechanical measurements and complement these results by biological assays for a more detailed toxicity mechanism [142].

HT microscopy aids in nanoparticle detection inside cells since nanoparticles are solid objects with RI values higher than most cellular components [143]. However, to avoid false interpretations on the entry of NMs in cells, special attention is needed to cellular structures (like chromatin which can surpass RI values higher than 1.39) with refractive indexes similar to NPs. For example, Liu et al. propose setting a limit for refractive index intensity, high enough for eliminating false-positive NPs interacting cells. However, this condition avoids visualizing cells with low amounts of NPs aggregates. Superparamagnetic iron oxides (SPIOs) have numerous biomedical applications, such as cancer treatment via hyperthermia [144]. To improve the outcome of this treatment, SPIOs need to be targeted into the cancer cells for further incorporation or fixing on the surface. As demonstrated by Frederich et al., HT microscopy is a suitable technique to study the incorporation of (SPIOs) into pancreatic cell lines, making simple the distinction between NPs bound to the cell membrane and fully incorporated NPs [138].

The application of NMs in the biomedical field arouses awareness to increase the efforts to evaluate NMs toxicity before their practical implementation. The application of NMs in the biomedical field is diverse. For instance, some NMs must be cytotoxic to cancerous cells, keeping specificity to avoid side effects in non-target cells. In vitro studies are fast and simple but sometimes not accurate to represent the interaction of NMs in a living organism. Due to their simplicity, it is desirable to implement in vitro models that mimic biological models more accurately and enable high throughput assessment. Searching for more representative in vitro models, a research group fabricated a 3D mini liver to reproduce closely physiological conditions. After, they evaluated the exposure of this model to different concentrations of nanodiamonds (NDs). They conducted toxicity assessment of NDs using traditional toxicological techniques and label-free microscopy (AFM and HTM). Holotomography results showed the internalization of the NDs inside cells. AFM analysis remarks changes in cell stiffness and cell membrane integrity. Holotomography and other cell viability assays demonstrated that NDs caused cytotoxicity and altered membrane integrity [145].

**Fig. 8:**
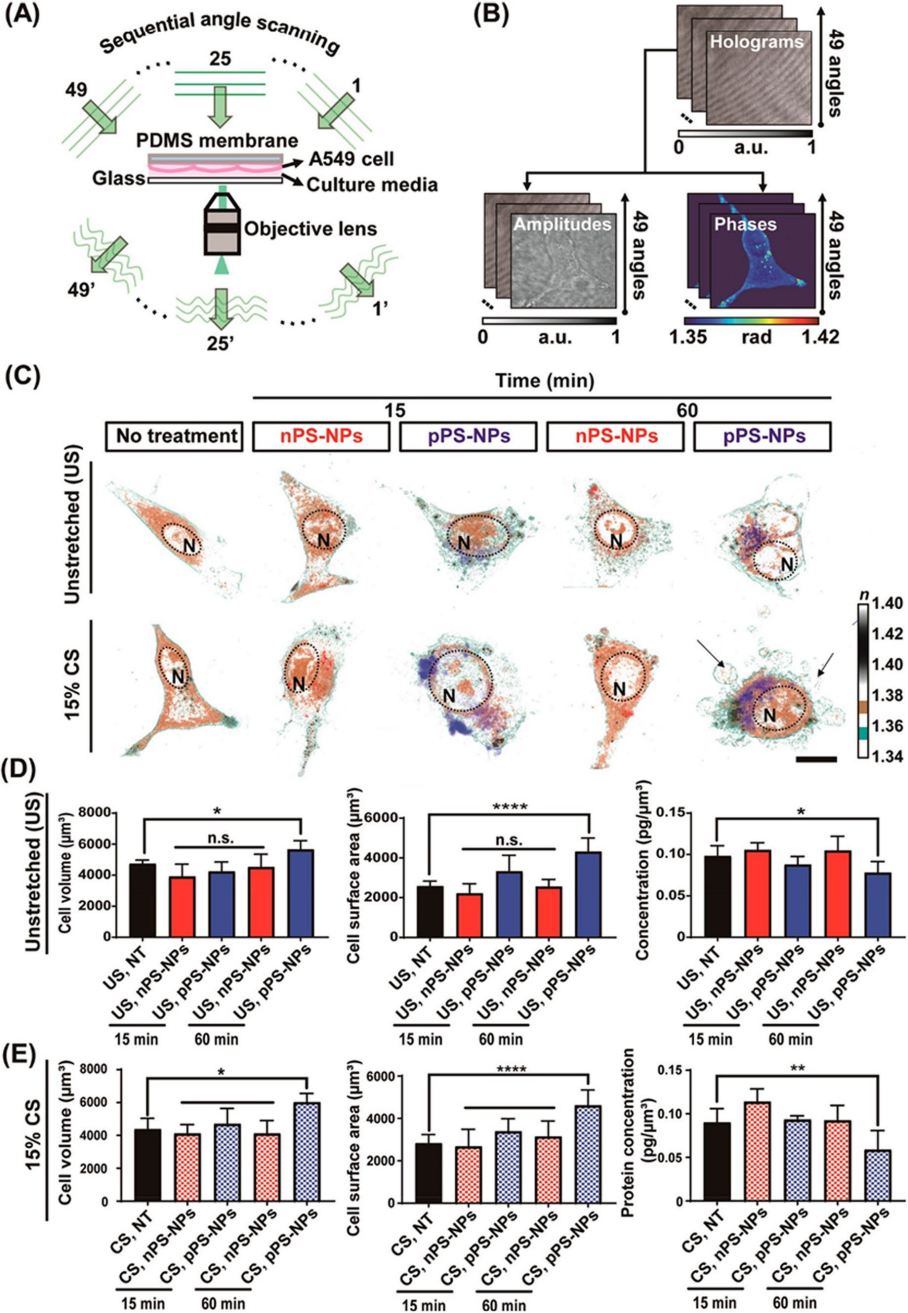
Live cell imaging of internalization of PS-NPs into alveola cells. **A** Scheme of RI acquisition via sequential angle scanning. **B** Integration of the amplitude and phase at 49 angles to reconstruct 3-D holograms. **C** 3-D RI tomographic images of nPS-NPs- and pPS-NPs-treated cells in two different conditions: unstretched and 15% CS at defined time points. A dotted circle indicates the nucleus (N) of a single cell. The blebbing phenotypes are marked with arrows. Scale bars: 100 μm. All experiments were performed in a TomoChamber (Tomocube) maintained at 37 °C and 5% CO_2_. **D**, **E** The estimation of cell volume, surface area, and protein concentration of a single cell (n = 5) under unstretched (US) and 15% CS, respectively. The color bar in the 3-D reconstruction images represents the 3-D rendered RI range [146]

Another example describes the evaluation of the toxicity of polystyrene (PS) NPs on alveolar cells in breath-mimicked conditions. One limitation of the in-vitro studies of alveolar cells is that cultured cells are static, missing the dynamic microenvironment of the human lungs. For more precise human pulmonary conditions, alveolar cells (A549) were deposited in flexible substrates (PDMS) and subjected to cyclic stretches under HT microscopy observations (Fig. 8). Under these conditions, the cells were exposed to PS-NPs (positively or negatively charged) to evaluate their cytotoxicity. Positively charged nanoparticles are more cytotoxic, causing swelling of the plasma membrane, further leading to apoptotic death. Also, more internalization of NPs occurs under cyclic stretches. Morphological changes and PS-NPs internalization into the cells was demonstrated using HT microscopy.

**Fig. 9:**
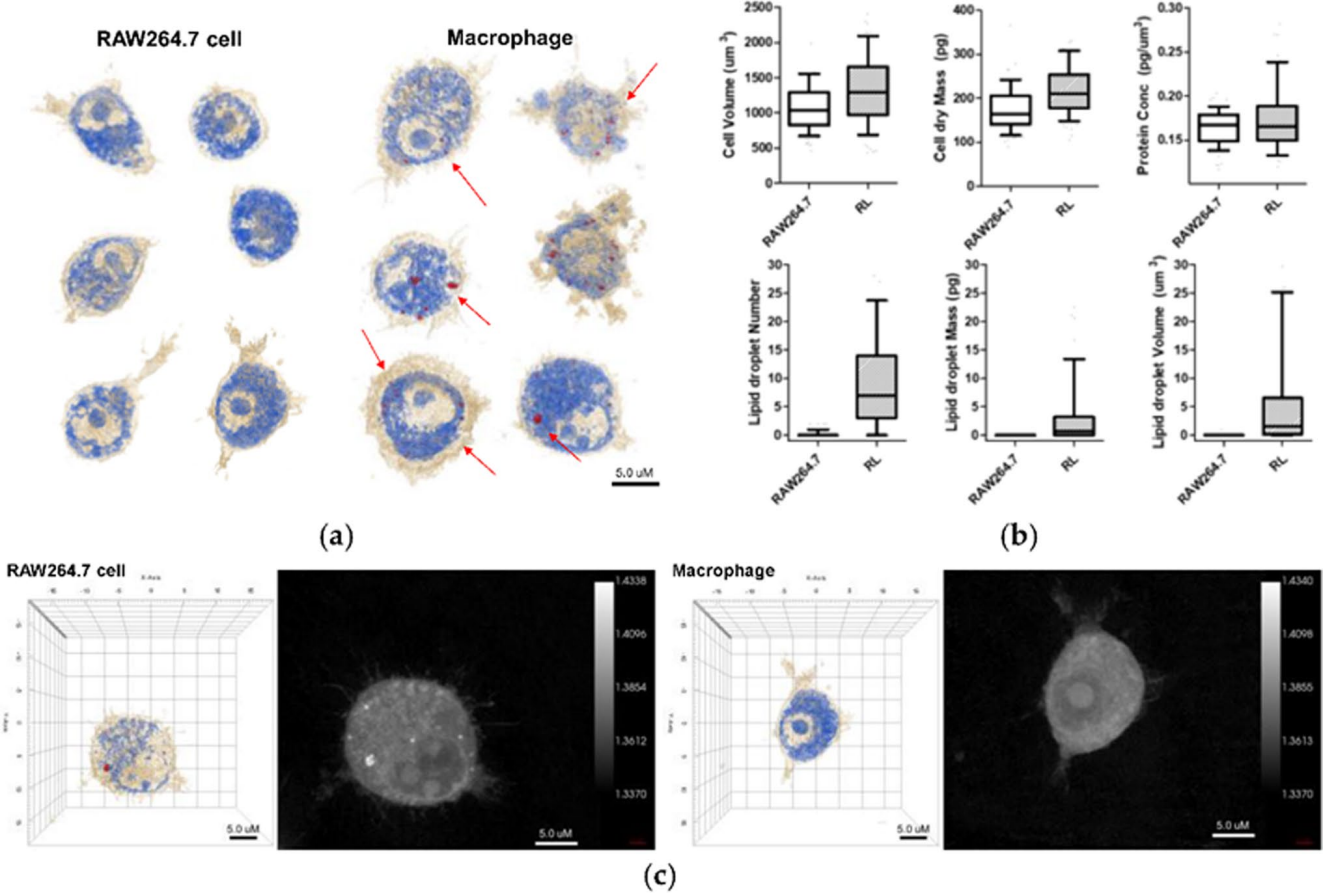
**a** 3D holotomographic images of LPS-induced macrophages show significant changes compared to RAW264.7 cell line. Cell size and morphological changes appeared after 48 h of incubation with LPS. **b** The characteristics of RAW264.7 cells and macrophages were quantitatively compared through numerical analysis. This analysis results exhibit the morphological and biological changes using RAW264.7 cell (n = 320) and macrophage (RL; n = 262). Data are presented as a box-and-whiskers plot, with 10–90 percentile of results. Additionally, all of the data show significant differences (p < 0.001). **c** 3D RI tomograms (left) of each cell were reconstructed from multiple 2D hologram images (right) [146]

A recent study evaluates the cytotoxicity and pro-inflammatory effects of WS_2_ and MoS_2_ in human bronchial cells (BEAS-2B cells), using classical toxicology assays and HT microscopy. Microscopy studies reveal changes in the size and shape of cells and loss of cell–cell contact due to exposure to NMs. They also expose a higher degree of cytoplasmic vacuolization, changes in the nucleus morphology, and intracellular location of mitochondria [147]. We previously discussed the importance of coupling different techniques to increase the potentialities of HT microscopy. Although 3D cell cultures reproduce more accurately in vivo physiological conditions, they are difficult to visualize by HT microscopy due to their thickness. Suematsu et al. [148] report the fabrication of ultrathin porous polymeric substrates, suitable for the microscopic study of 3D cultures of cells. Nowadays, it is possible the use of HT microscopy for organoid imaging. Lately, NMs find a use for controlled-drug delivery. The use of macrophages as drug delivery agents (Trojan horse) is popular to avoid immune system activation. A recent study explores loading Au NPs into macrophages for targeted drug delivery. HT microscopy reveals the distribution of Au NPs in the cells [149]. Following this same approach, Kang et al. use HT microscopy to decipher the interaction between NPs and immune cells. They observe an increase in lipid droplets and cell volume after NP internalization (Fig. 9). They also discuss that loading Au NPs into alveolar macrophages is suitable strategy of drug delivery for asthma [150].

Biological auto luminescence (BAL) serves as a reference to evaluate the healthiness of living organisms. However, BAL luminescence intensity values are low. NPs are suitable for the enhancement of BAL. Sardarabadi et al. [151] designed mito-liposomal gold nanocarriers; holotomography revealed no changes in cell morphology before cell internalization and an increased affinity to the mitochondria, an essential organelle involved in BAL processes, thus helping to increase BAL measurement in U2OS cells. To our knowledge, not many studies report on using AFM and HTM as complementary techniques to monitor the entry, transformation, and final destination of NMs in living cells. With AFM imaging is limited the possibility of looking inside the cell; whereas HTM imaging clearly illustrates the entry of NMs in the cells [152].

Numerous previous studies discuss the advantages of integrating imaging techniques to obtain more information on a complex biological system [46, 77, 80]. Preliminary studies on correlative AFM-HTM remark on the convenience of applying integrated sample analysis. Before the existence of commercial HTM instruments, researchers investigated the outcomes of analyzing samples by correlative AFM and Digital Holographic Microscopy (DHM) [98]. AFM is a high-resolution scanning method that requires high acquisition time, a limitation solved by the fast rate of DHM acquisition. On the other hand, DHM is sensitive to changes in RI within physical thickness, limiting its traverse resolution. It is possible to solve this limitation by the co-integration of DHM and AFM since AFM imaging provides higher traverse resolution at the nanometer scale.

To our knowledge, there are scarce studies regarding AFM and HTM as correlative techniques for biomedical investigation. The sequential use of these techniques results in a precise identification of different cellular organelles, super-resolution morphometric analysis, and study of the dynamics of biological processes. A late study shows a detailed investigation (that correlates traditional toxicity assays with AFM and HTM analysis) of the bioactivity of aluminum-doped zinc oxide using SH-SY5Y cells as biological model organisms [4].

**Fig. 10:**
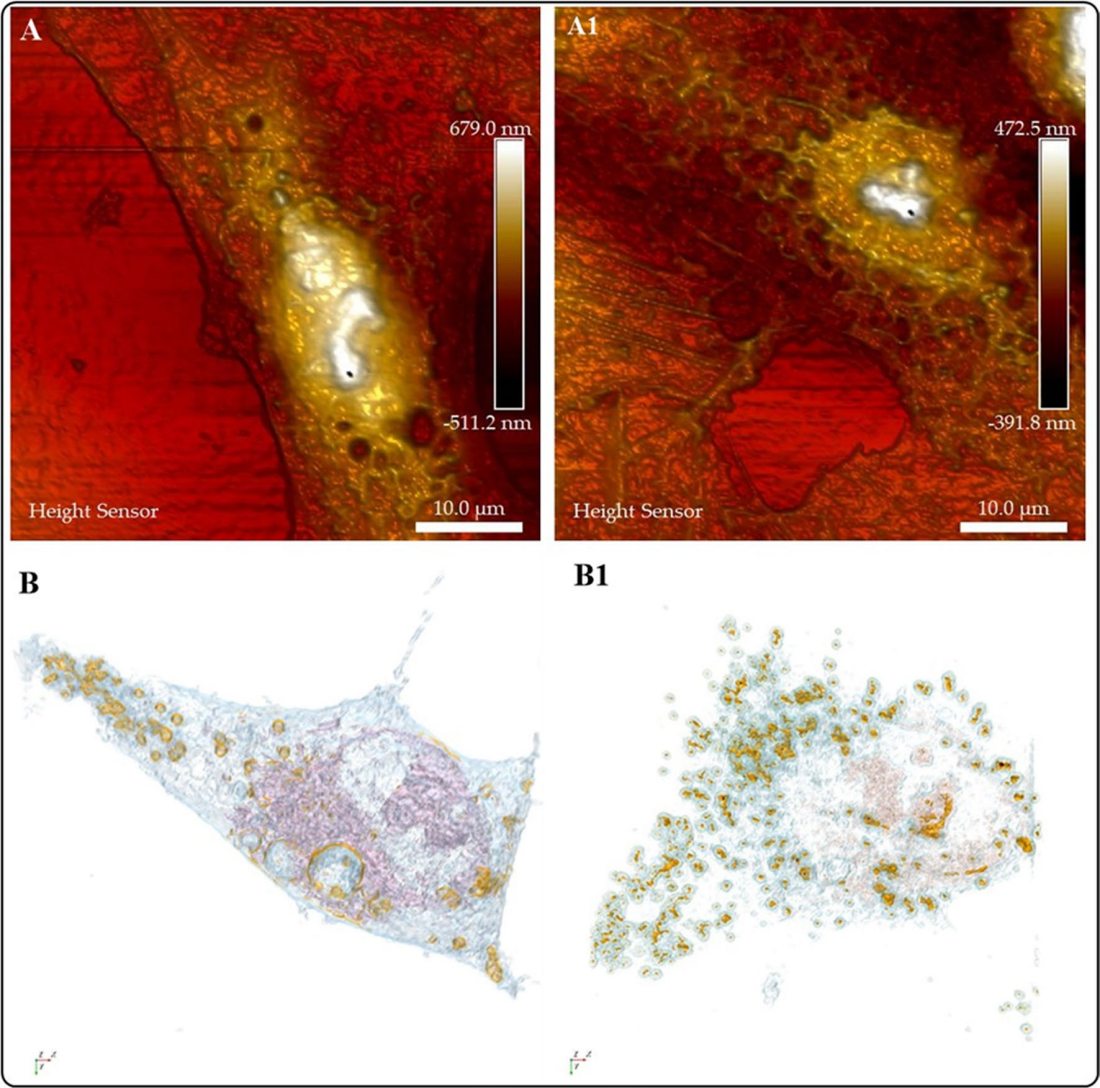
Schematic describing the interaction of Au NPs with A549 cells. The changes in morphology are consistent by imaging with AFM and HT microscopy. (**A**) AFM analysis of A549 cell (control group), illustrating the typical morphological characteristics of this cell line; (**A1**) AFM analysis of A549 cell upon interaction with Au NPs, showing changes in the cell morphology; (**B**) HTM analysis of A549 cell (control group), illustrating the typical morphological characteristics of this cell line. In addition, HTM images show an increase in lipid drop production and internalization of Au NMs in the organelle to detoxification (**B1**)

To this point, it is evident that due to their versatility, researchers should consider these microscopies as a routine tool to study the complex interactions of NMs in diverse cells or tissues (Fig. 10). The data generated by these techniques is complementary because, despite the high-resolution surface images rendered by AFM, it is limited the possibility of looking inside the cell.; whereas HTM imaging clearly illustrates the entry of NMs in the cells. Figure 10 shows AFM (Fig. 10A and A1) and HTM (Figs. 10B and B1) images that depict the changes in A549 cells treated with AuNMs (Fig. 10A and A1). In addition to the morphological changes seen in HTM images, it is also possible to see an increase in the lipid droplet production and internalization of Au NMs in this organelle to clear NMs. This knowledge can contribute to safer nanomedicine developments.

## Conclusions and prospects

This review highlights the versatility of AFM and HTM microscopies as valuable tools in biomedical research that provide real-time, label-free, and high-resolution images of living cells under different conditions. AFM and HTM can generate data not just on the morphology of the cells but also on mechanical, magnetic, electrochemical properties, or dry mass composition. Recent studies remark on the advantages of using these techniques in biomedical-related fields. AFM microscopy offers plenty of alternatives for the multiparametric and multifunctional characterization of biological systems with improved performance: fast scanning, fast force spectroscopy analysis, sub-piconewton force precision, thermal stability, ultra-low noise, and user-friendly for researchers at all levels of experience. The integration of AFM with another microscopy (inverted optical microscope) or spectroscopy techniques (Raman, IR) produces correlated measurements that offer a more comprehensive understanding (dynamics, structural, mechanical, chemical, and functional heterogeneity) of complex biological systems. Latest developments allow coupling AFM microscope to a picobalance to monitor time-dependent mass measurements in physiological conditions. Modern complex AFM systems (AFM-picobalance-inverted optical microscope) compute data to link cell mass dynamics to cell morphology and growth with application in the study of a vast number of cellular processes: Cell mass or volume regulation, cell migration, cell nutrition, cell division, cell cycle progression, fat cell storage, viral infection-related mechanisms, and new therapies for cancer, among others. HT microscopy imaging is appropriate for distinguishing neoplastic changes with sub-cell determination in vivo as a non-invasive technique (without the requirement for biopsy). HT microscopy is a simple and robust strategy for the diagnosis, monitoring, and elucidation of the mechanisms of disease development. It is also suitable for research studies in cytotoxicity (phenotypic screening of living cells, cell responses to drug interaction, dose-dependent cell death), and cell metabolism. AFM and HTM microscopies as standard strategies in biological or biomedical research enables researchers to obtain more precise information about cells, organelles, and their interactions with exogenous agents. This knowledge is crucial to addressing challenging burdens in biomedical research and drug discovery.

## Data Availability

Not applicable.

## References

[1] Lemon WC, McDole K (2020). Live-cell imaging in the era of too many microscopes. Curr Opin Cell Biol.

[2] Proa-Coronado S, Séverac C, Martinez-Rivas A, Dague E (2020). Beyond the paradigm of nanomechanical measurements on cells using AFM: an automated methodology to rapidly analyse thousands of cells. Nanoscale Horiz.

[3] Li M, Xi N, Liu L (2021). Peak force tapping atomic force microscopy for advancing cell and molecular biology. Nanoscale.

[4] Jimenez-chavez A, Pedroza-herrera G, Betancourt-reyes I, Ruiz ADV, Masuoka-ito D, Zapien JA, Medina-ramirez IE (2024). Aluminum enhances the oxidative damage of ZnO NMs in the human. Discov Nano.

[5] Hernandez R, Jimenez-Chávez A, De Vizcaya A, Lozano-Alvarez JA, Esquivel K, Medina-Ramírez IE (2023). Synthesis of TiO_2_-Cu^2+^/CuI nanocomposites and evaluation of antifungal and cytotoxic activity. Nanomaterials.

[6] Medina-Ramírez IE, Marroquin-Zamudio A, Martínez-Montelongo JH, Romo-Lozano Y, Zapien JA, Perez-Larios A (2022). Enhanced photocatalytic and antifungal activity of ZnO–Cu^2+^ and Ag@ZnO–Cu^2+^ materials. Ceram Int.

[7] Singh AV, Varma M, Laux P, Choudhary S, Datusalia AK, Gupta N, Luch A, Gandhi A, Kulkarni P, Nath B (2023). Artificial intelligence and machine learning disciplines with the potential to improve the nanotoxicology and nanomedicine fields: a comprehensive review. Arch Toxicol.

[8] Kim D, Lee S, Lee M, Oh J, Yang SA, Park YK. Holotomography: refractive index as an intrinsic imaging contrast for 3-D label-free live cell imaging. In: Adv. Exp. Med. Biol. 2021, pp. 211–238. 10.1007/978-981-33-6064-8_10. 10.1007/978-981-33-6064-8_10 33834439

[9] Choi J, Kim H-J, Sim G, Lee S, Park WS, Park JH, Kang H-Y, Lee M, Do-Heo W, Choo J, Min H, Park Y (2021). Label-free three-dimensional analyses of live cells with deep-learning-based segmentation exploiting refractive index distributions. BioRxiv.

[10] Park YK, Depeursinge C, Popescu G (2018). Quantitative phase imaging in biomedicine. Nat Photonics.

[11] Kim SY, Lee JH, Shin Y, Kim TK, Won Lee J, Pyo MJ, Lee AR, Pack CG, Cho YS (2022). Label-free imaging and evaluation of characteristic properties of asthma-derived eosinophils using optical diffraction tomography. Biochem Biophys Res Commun.

[12] Kim TK, Lee BW, Fujii F, Kim JK, Pack CG (2019). Physicochemical properties of nucleoli in live cells analyzed by label-free optical diffraction tomography. Cells.

[13] Meyer RA (1979). Light scattering from biological cells: dependence of backscatter radiation on membrane thickness and refractive index. Appl Opt.

[14] Chamot S, Migacheva E, Seydoux O, Marquet P, Depeursinge C (2010). Physical interpretation of the phase function related parameter γ studied with a fractal distribution of spherical scatterers. Opt Express.

[15] Zhang Q, Zhong L, Tang P, Yuan Y, Liu S, Tian J, Lu X (2017). Quantitative refractive index distribution of single cell by combining phase-shifting interferometry and AFM imaging. Sci Rep.

[16] Liu PY, Chin LK, Ser W, Chen HF, Hsieh CM, Lee CH, Sung KB, Ayi TC, Yap PH, Liedberg B, Wang K, Bourouina T, Leprince-Wang Y (2016). Cell refractive index for cell biology and disease diagnosis: past, present and future. Lab Chip.

[17] Sandoz PA, Tremblay C, Equis S, Pop S, Pollaro L, Cotte Y, van der Goot FG, Frechin M. Label free 3D analysis of organelles in living cells by refractive index shows pre-mitotic organelle spinning in mammalian stem cells. BioRxiv 2018;407239.

[18] Moreno H, Archetti L, Gibbin E, Grandchamp AE, Fréchin M (2021). Artificial intelligence-powered automated holotomographic microscopy enables label-free quantitative biology. Micros Today.

[19] Sandoz PA, Tremblay C, Gisou van der Goot F, Frechin M (2019). Image-based analysis of living mammalian cells using label-free 3D refractive index maps reveals new organelle dynamics and dry mass flux. PLoS Biol.

[20] D’Brant LY, Desta H, Khoo TC, Sharikova AV, Mahajan SD, Khmaladze A (2019). Methamphetamine-induced apoptosis in glial cells examined under marker-free imaging modalities. J Biomed Opt.

[21] Pollaro L, Dalla Piazza B, Cotte Y (2015). Digital staining: microscopy of live cells without invasive chemicals. Micros Today.

[22] Lambert A (2020). Live cell imaging with holotomography and fluorescence. Micros Today.

[23] Park S, Lee LE, Kim H, Kim JE, Lee SJ, Yoon S, Shin S, Kang H, Park YK, Song JJ, Lee S (2021). Detection of intracellular monosodium urate crystals in gout synovial fluid using optical diffraction tomography. Sci Rep.

[24] He Y, Zhou N, Ziemczonok M, Wang Y, Lei L, Duan L, Zhou R (2023). Standardizing image assessment in optical diffraction tomography. Opt Lett.

[25] Kang S, Zhou R, Brelen M, Mak HK, So PTC, Yaqoob Z. Reflection-mode optical diffraction tomography for label-free imaging of thick biological specimens; 2022. Preprint at http://arxiv.org/abs/2202.13668

[26] Lee D, Lee M, Kwak H, Kim YS, Shim J, Jung JH, Park W, Park J-H, Lee S, Park Y (2022). High-fidelity optical diffraction tomography of live organisms using iodixanol refractive index matching. Biomed Opt Express.

[27] Jo YJ, Cho H, Lee SY, Choi G, Kim G, Min HS, Park YK (2018). Quantitative phase imaging and artificial intelligence: a review. IEEE J Sel Top Quantum Electron.

[28] Shevkunov I, Ziemczonok M, Kujawińska M, Egiazarian K (2022). Complex-domain SVD- and sparsity-based denoising for optical diffraction tomography. Opt Lasers Eng.

[29] Ziemczonok M, Kuś A, Kujawińska M (2022). Optical diffraction tomography meets metrology—measurement accuracy on cellular and subcellular level. Meas J Int Meas Confed.

[30] Srichana T, Thawithong E, Nakpheng T, Paul PK (2023). Flow cytometric analysis, confocal laser scanning microscopic, and holotomographic imaging demonstrate potentials of levofloxacin dry powder aerosols for TB treatment. J Drug Deliv Sci Technol.

[31] Salucci S, Battistelli M, Burattini S, Sbrana F, Falcieri E (2020). Holotomographic microscopy: a new approach to detect apoptotic cell features. Microsc Res Tech.

[32] Koutsogiannis Z, Mina JGM, Suman R, Denny PW (2023). Assessment of *Toxoplasma gondii* lytic cycle and the impact of a gene deletion using 3D label-free optical diffraction holotomography. Front Cell Infect Microbiol.

[33] Ryu DH, Nam H, Jeon JS, Park YK (2021). Reagent- and actuator-free analysis of individual erythrocytes using three-dimensional quantitative phase imaging and capillary microfluidics. Sens Actuators B Chem.

[34] Ali A, Abouleila Y, Amer S, Furushima R, Emara S, Equis S, Cotte Y, Masujima T (2016). Quantitative live single-cell mass spectrometry with spatial evaluation by three-dimensional holographic and tomographic laser microscopy. Anal Sci.

[35] Zhao J, Matlock A, Zhu H, Song Z, Zhu J, Wang B, Chen F, Zhan Y, Chen Z, Xu Y, Lin X, Tian L, Cheng JX (2022). Bond-selective intensity diffraction tomography. Nat Commun.

[36] Baczewska M, Eder K, Ketelhut S, Kemper B, Kujawińska M (2021). Refractive index changes of cells and cellular compartments upon paraformaldehyde fixation acquired by tomographic phase microscopy. Cytom Part A.

[37] Park D, Lee D, Kim Y, Park Y, Lee YJ, Lee JE, Yeo MK, Kang MW, Chong Y, Han SJ, Choi J, Park JE, Koh Y, Lee J, Park YK, Kim R, Lee JS, Choi J, Lee SH, Ku B, Kang DH, Chung C (2023). Cryobiopsy: a breakthrough strategy for clinical utilization of lung cancer organoids. Cells.

[38] Pollaro L, Equis S, Dalla Piazza B, Cotte Y (2016). Stain-free 3D nanoscopy of living cells. Opt Photonik.

[39] Jiang H, Woo Kwon J, Lee S, Jo YJ, Namgoong S, Rui Yao X, Yuan B, Bao Zhang J, Park YK, Kim NH (2019). Reconstruction of bovine spermatozoa substances distribution and morphological differences between Holstein and Korean native cattle using three-dimensional refractive index tomography. Sci Rep.

[40] Kreplak L (2016). Introduction to atomic force microscopy (AFM) in biology. Curr Protoc Protein Sci.

[41] Dufrêne YF, Ando T, Garcia R, Alsteens D, Martinez-Martin D, Engel A, Gerber C, Müller DJ (2017). Imaging modes of atomic force microscopy for application in molecular and cell biology. Nat Nanotechnol.

[42] Miranda A, Gómez-Varela AI, Stylianou A, Hirvonen LM, Sánchez H, De Beule PAA (2021). How did correlative atomic force microscopy and super-resolution microscopy evolve in the quest for unravelling enigmas in biology?. Nanoscale.

[43] Maver U, Velnar T, Gaberšček M, Planinšek O, Finšgar M (2016). Recent progressive use of atomic force microscopy in biomedical applications. Trends Anal Chem.

[44] Uchihashi T, Ganser C (2020). Recent advances in bioimaging with high-speed atomic force microscopy. Biophys Rev.

[45] dos Santos ACVD, Hondl N, Ramos-Garcia V, Kuligowski J, Lendl B, Ramer G (2023). AFM-IR for nanoscale chemical characterization in life sciences: recent developments and future directions. ACS Meas Sci Au.

[46] Shi X, Qing W, Marhaba T, Zhang W (2020). Atomic force microscopy—scanning electrochemical microscopy (AFM-SECM) for nanoscale topographical and electrochemical characterization: principles, applications and perspectives. Electrochim Acta.

[47] Xia F, Youcef-Toumi K (2022). Review: advanced atomic force microscopy modes for biomedical research. Biosensors.

[48] Krull A, Hirsch P, Rother C, Schiffrin A, Krull C (2020). Artificial-intelligence-driven scanning probe microscopy. Commun Phys.

[49] Houhou R, Bocklitz T (2021). Trends in artificial intelligence, machine learning, and chemometrics applied to chemical data. Anal Sci Adv.

[50] Parot P, Dufrêne YF, Hinterdorfer P, Le Grimellec C, Navajas D, Pellequer JL, Scheuring S (2007). Past, present and future of atomic force microscopy in life sciences and medicine. J Mol Recognit.

[51] Bhat SV, Price JDW, Dahms TES (2021). AFM-based correlative microscopy illuminates human pathogens. Front Cell Infect Microbiol.

[52] Zhou L, Cai M, Tong T, Wang H (2017). Progress in the correlative atomic force microscopy and optical microscopy. Sensors.

[53] Kubota R, Tanaka W, Hamachi I (2021). Microscopic imaging techniques for molecular assemblies: electron, atomic force, and confocal microscopies. Chem Rev.

[54] Uchihashi T, Watanabe H, Fukuda S, Shibata M, Ando T (2016). Functional extension of high-speed AFM for wider biological applications. Ultramicroscopy.

[55] Dufrêne YF (2004). Using nanotechniques to explore microbial surfaces. Nat Rev Microbiol.

[56] Dufrêne YF (2002). Atomic force microscopy, a powerful tool in microbiology. J Bacteriol.

[57] Formosa-Dague C, Duval RE, Dague E (2018). Cell biology of microbes and pharmacology of antimicrobial drugs explored by atomic force microscopy. Semin Cell Dev Biol.

[58] Lin YC, Huang C, Lai HC (2019). Revealing the ultrastructure of the membrane pores of intact Serratia marcescens cells by atomic force microscopy. Heliyon.

[59] Martínez-Montelongo JH, Medina-Ramírez IE, Romo-Lozano Y, Zapien JA (2020). Development of a sustainable photocatalytic process for air purification. Chemosphere.

[60] Efremov YM, Suter DM, Timashev PS, Raman A (2022). 3D nanomechanical mapping of subcellular and sub-nuclear structures of living cells by multi-harmonic AFM with long-tip microcantilevers. Sci Rep.

[61] Medina-Ramírez IE, Díaz de León-Macias CE, Pedroza-Herrera G, Gonzáles-Segovia R, Zapien JA, Rodríguez-López JL (2020). Evaluation of the biocompatibility and growth inhibition of bacterial biofilms by ZnO, Fe_3_O_4_ and ZnO@Fe_3_O_4_ photocatalytic magnetic materials. Ceram Int.

[62] Medina-Ramírez IE, Díaz de León Olmos MA, Muñoz Ortega MH, Zapien JA, Betancourt I, Santoyo-Elvira N (2020). Development and assessment of nano-technologies for cancer treatment: cytotoxicity and hyperthermia laboratory studies. Cancer Invest.

[63] Jansen KA, Donato DM, Balcioglu HE, Schmidt T, Danen EHJ, Koenderink GH (1853). A guide to mechanobiology: where biology and physics meet. Biochim Biophys Acta Mol Cell Res.

[64] Krieg M, Fläschner G, Alsteens D, Gaub BM, Roos WH, Wuite GJL, Gaub HE, Gerber C, Dufrêne YF, Müller DJ (2019). Atomic force microscopy-based mechanobiology. Nat Rev Phys.

[65] Kasas S, Stupar P, Dietler G (2018). AFM contribution to unveil pro- and eukaryotic cell mechanical properties. Appl At Force Microsc Cell Biol.

[66] Guillaume-Gentil O, Potthoff E, Ossola D, Franz CM, Zambelli T, Vorholt JA (2014). Force-controlled manipulation of single cells: from AFM to FluidFM. Trends Biotechnol.

[67] Miyazawa K, Penedo M, Furusho H, Ichikawa T, Alam MS, Miyata K, Nakamura C, Fukuma T (2023). Nanoendoscopy-AFM for visualizing intracellular nanostructures of living cells. Microsc Microanal.

[68] Ichikawa T, Alam MS, Penedo M, Matsumoto K, Fujita S, Miyazawa K, Furusho H, Miyata K, Nakamura C, Fukuma T (2023). Protocol for live imaging of intracellular nanoscale structures using atomic force microscopy with nanoneedle probes. STAR Protoc.

[69] Fukuma T (2024). Visualizing the inside of three-dimensional self-organizing systems by three-dimensional atomic force microscopy. Jpn J Appl Phys.

[70] Ando T (2018). High-speed atomic force microscopy and its future prospects. Biophys Rev.

[71] Ando T, Uchihashi T, Scheuring S (2014). Filming biomolecular processes by high-speed atomic force microscopy. Chem Rev.

[72] Dufrêne YF, Viljoen A, Mignolet J, Mathelié-Guinlet M (2021). AFM in cellular and molecular microbiology. Cell Microbiol.

[73] Schoenwald K, Peng ZC, Noga D, Qiu SR, Sulchek T (2010). Integration of atomic force microscopy and a microfluidic liquid cell for aqueous imaging and force spectroscopy. Rev Sci Instrum.

[74] Efremov YM, Okajima T, Raman A (2019). Measuring viscoelasticity of soft biological samples using atomic force microscopy. Soft Matter.

[75] Dazzi A, Prater CB (2017). AFM-IR: technology and applications in nanoscale infrared spectroscopy and chemical imaging. Chem Rev.

[76] Mathurin J, Deniset-Besseau A, Bazin D, Dartois E, Wagner M, Dazzi A (2022). Photothermal AFM-IR spectroscopy and imaging: status, challenges, and trends. J Appl Phys.

[77] Geisse NA (2009). AFM and combined optical techniques. Mater Today.

[78] Colombo F, Norton EG, Cocucci E (2021). Microscopy approaches to study extracellular vesicles. Biochim Biophys Acta Gen Subj.

[79] Cascione M, de Matteis V, Rinaldi R, Leporatti S (2017). Atomic force microscopy combined with optical microscopy for cells investigation. Microsc Res Tech.

[80] Staunton JR, Doss BL, Lindsay S, Ros R (2016). Correlating confocal microscopy and atomic force indentation reveals metastatic cancer cells stiffen during invasion into collagen i matrices. Sci Rep.

[81] Bagheri AR, Aramesh N, Bilal M, Xiao J, Kim HW, Yan B (2021). Carbon nanomaterials as emerging nanotherapeutic platforms to tackle the rising tide of cancer—A review. Bioorganic Med Chem.

[82] Deng X, Xiong F, Li X, Xiang B, Li Z, Wu X, Guo C, Li X, Li Y, Li G, Xiong W, Zeng Z (2018). Application of atomic force microscopy in cancer research. J Nanobiotechnology.

[83] Stylianou A, Lekka M, Stylianopoulos T (2018). AFM assessing of nanomechanical fingerprints for cancer early diagnosis and classification: from single cell to tissue level. Nanoscale.

[84] Di Santo R, Romanò S, Mazzini A, Jovanović S, Nocca G, Campi G, Papi M, De Spirito M, Di Giacinto F, Ciasca G (2021). Recent advances in the label-free characterization of exosomes for cancer liquid biopsy: from scattering and spectroscopy to nanoindentation and nanodevices. Nanomaterials.

[85] Kubiak A, Zieliński T, Pabijan J, Lekka M (2020). Nanomechanics in monitoring the effectiveness of drugs targeting the cancer cell cytoskeleton. Int J Mol Sci.

[86] Zhang H, Xiao L, Li Q, Qi X, Zhou A (2018). Microfluidic chip for non-invasive analysis of tumor cells interaction with anti-cancer drug doxorubicin by AFM and Raman spectroscopy. Biomicrofluidics.

[87] Andrei L, Kasas S, Ochoa Garrido I, Stanković T, Suárez Korsnes M, Vaclavikova R, Assaraf YG, Pešić M (2020). Advanced technological tools to study multidrug resistance in cancer. Drug Resist Updat.

[88] Szlasa W, Supplitt S, Drąg-Zalesińska M, Przystupski D, Kotowski K, Szewczyk A, Kasperkiewicz P, Saczko J, Kulbacka J (2020). Effects of curcumin based PDT on the viability and the organization of actin in melanotic (A375) and amelanotic melanoma (C32)—in vitro studies. Biomed Pharmacother.

[89] Łapińska Z, Dębiński M, Szewczyk A, Choromańska A, Kulbacka J, Saczko J (2021). Electrochemotherapy with calcium chloride and 17β-estradiol modulated viability and apoptosis pathway in human ovarian cancer. Pharmaceutics.

[90] Paidi SK, Shah V, Raj P, Glunde K, Pandey R, Barman I (2021). Coarse Raman and optical diffraction tomographic imaging enable label-free phenotyping of isogenic breast cancer cells of varying metastatic potential. Biosens Bioelectron.

[91] Nissim N, Dudaie M, Barnea I, Shaked NT (2021). Real-time stain-free classification of cancer cells and blood cells using interferometric phase microscopy and machine learning. Cytom Part A.

[92] Palacios-Acedo AL, Mezouar S, Mège D, Crescence L, Dubois C, Panicot-Dubois L (2021). P2RY12-inhibitors reduce cancer-associated thrombosis and tumor growth in pancreatic cancers. Front Oncol.

[93] Szlasa W, Kiełbik A, Szewczyk A, Rembiałkowska N, Novickij V, Tarek M, Saczko J, Kulbacka J (2021). Oxidative effects during irreversible electroporation of melanoma cells-in vitro study. Molecules.

[94] Lee KP, Baek S, Yoon MS, Park JS, Hong BS, Lee SJ, Oh SJ, Kwon SH, Lee R, Lee DH, Park KS, Moon BS (2022). Potential anticancer effect of aspirin and 2′-hydroxy-2,3,′-trimethoxychalcone-linked polymeric micelles against cervical cancer through apoptosis. Oncol Lett.

[95] Zhu X, Shen H, Yin X, Long L, Xie C, Liu Y, Hui L, Lin X, Fang Y, Cao Y, Xu Y, Li M, Xu W, Li Y (2016). MiR-186 regulation of Twist1 and ovarian cancer sensitivity to cisplatin. Oncogene.

[96] Xin L, Xiao W, Che L, Liu J, Miccio L, Bianco V, Memmolo P, Ferraro P, Li X, Pan F (2021). Label-free assessment of the drug resistance of epithelial ovarian cancer cells in a microfluidic holographic flow cytometer boosted through machine learning. ACS Omega.

[97] Aldonza MBD, Reyes RDD, Kim YS, Ku J, Barsallo AM, Hong JY, Lee SK, Ryu HS, Park YK, Cho JY, Kim Y (2021). Chemotherapy confers a conserved secondary tolerance to EGFR inhibition via AXL-mediated signaling bypass. Sci Rep.

[98] Cardenas N, Ingle N, Yu L, Mohanty S (2011). Development of a digital holographic microscopy system integrated with atomic force microscope, three-dimensional multidimens. Microsc Image Acquis Process XVIII.

[99] Villalba MI, Venturelli L, Arnal L, Masson C, Dietler G, Vela ME, Yantorno O, Kasas S (2022). Effect of antibiotics on mechanical properties of *Bordetella pertussis* examined by atomic force microscopy. Micron.

[100] Zdarta A, Kaczorek E (2023). Nanomechanical changes in probiotic bacteria under antibiotics exposure: implications on Lactobacillus biofilm formation. Biochim Biophys Acta Mol Cell Res.

[101] Yamashita H, Taoka A, Uchihashi T, Asano T, Ando T, Fukumori Y (2012). Single-molecule imaging on living bacterial cell surface by high-speed AFM. J Mol Biol.

[102] Jiang Y, Yuan Z, Huang J (2020). Substituted hydroxyapatite: a recent development. Mater Technol.

[103] Backes EH, Pires LDN, Beatrice CAG, Costa LC, Passador FR, Pessan LA (2020). Fabrication of biocompatible composites of poly(lactic acid)/hydroxyapatite envisioning medical applications. Polym Eng Sci.

[104] Ungureanu E, Vladescu A, Parau AC, Mitran V, Cimpean A, Tarcolea M, Vranceanu DM, Cotrut CM (2023). In vitro evaluation of Ag- and Sr-doped hydroxyapatite coatings for medical applications. Materials.

[105] Xiao Y, Cheng Y, He P, Wu X, Li Z (2021). New insights into external layers of cyanobacteria and microalgae based on multiscale analysis of AFM force-distance curves. Sci Total Environ.

[106] Zhao LS, Su HN, Li K, Bin Xie B, Liu LN, Zhang XY, Chen XL, Huang F, Zhou BC, Zhang YZ (2016). Supramolecular architecture of photosynthetic membrane in red algae in response to nitrogen starvation. Biochim Biophys Acta Bioenerg.

[107] Atomic H, Microscopy F, Kobayashi K, Kodera N, Kasai T, Tahara YO, Toyonaga T (2021). Movements of mycoplasma mobile gliding machinery detected. MBio.

[108] Kikuchi Y, Obana N, Toyofuku M, Kodera N, Soma T, Ando T, Fukumori Y, Nomura N, Taoka A (2020). Diversity of physical properties of bacterial extracellular membrane vesicles revealed through atomic force microscopy phase imaging. Nanoscale.

[109] Demir-Yilmaz I, Guiraud P, Formosa-Dague C (2021). The contribution of atomic force microscopy (AFM) in microalgae studies: a review. Algal Res.

[110] Mignolet J, Dufre YF (2021). AFM force-clamp spectroscopy captures the nanomechanics of the Tad pilus retraction. Nanoscale Horizons.

[111] Chantraine C, Mathelié-Guinlet M, Pietrocola G, Speziale P, Dufrêne YF (2021). AFM identifies a protein complex involved in pathogen adhesion which ruptures at three nanonewtons. Nano Lett.

[112] Viela F, Alfeo MJ, Pietrocola G, Speziale P, Mathelie M (2020). Single-molecule analysis demonstrates stress-enhanced binding between *Staphylococcus aureus* surface protein IsdB and host cell integrins ´. Nano Lett.

[113] Kim G, Ahn D, Kang M, Jo Y, Ryu D, Kim H, Song J, Ryu JS, Choi G, Chung HJ, Kim K, Chung DR, Yoo IY, Huh HJ, Min H, Lee NY, Park Y (2019). Rapid and label-free identification of individual bacterial pathogens exploiting three-dimensional quantitative phase imaging and deep learning. BioRxiv.

[114] Oh J, Ryu JS, Lee M, Jung J, Han S, Chung HJ, Park Y (2020). Three-dimensional label-free observation of individual bacteria upon antibiotic treatment using optical diffraction tomography. Biomed Opt Express.

[115] Kim M, Cheon Y, Shin D, Choi J, Nielsen JE, Jeong MS, Nam HY, Kim SH, Lund R, Jenssen H, Barron AE, Lee S, Seo J (2023). Real-Time monitoring of multitarget antimicrobial mechanisms of peptoids using label-free imaging with optical diffraction tomography. Adv Sci.

[116] Jo Y, Park S, Jung J, Yoon J, Joo H, Kim M, Kang S-J, Choi MC, Lee SY, Park Y (2017). Holographic deep learning for rapid optical screening of anthrax spores. Sci Adv.

[117] Larrazabal C, Silva LMR, Hermosilla C, Taubert A (2021). Ezetimibe blocks *Toxoplasma gondii*-, *Neospora caninum*- And *Besnoitia besnoiti*-tachyzoite infectivity and replication in primary bovine endothelial host cells. Parasitology.

[118] Lopez-Osorio S, Velasquez ZD, Conejeros I, Taubert A, Hermosilla C (2021). Morphometric analysis of aerobic *Eimeria bovis* sporogony using live cell 3D holotomographic microscopy imaging. Parasitol Res.

[119] López-Osorio S, Silva LMR, Chaparro-Gutierréz JJ, Velásquez ZD, Taubert A, Hermosilla C (2020). Optimized excystation protocol for ruminant *Eimeria bovis*- and *Eimeria arloingi*-sporulated oocysts and first 3D holotomographic microscopy analysis of differing sporozoite egress. Parasitol Int.

[120] Firdaus ER, Park JH, Lee SK, Park YK, Cha GH, Han ET (2020). 3D morphological and biophysical changes in a single tachyzoite and its infected cells using three-dimensional quantitative phase imaging. J Biophotonics.

[121] Velásquez ZD, Lopez-Osorio S, Pervizaj-Oruqaj L, Herold S, Hermosilla C, Taubert A (2020). *Besnoitia besnoiti*–driven endothelial host cell cycle alteration. Parasitol Res.

[122] Zhou E, Silva LMR, Conejeros I, Velásquez ZD, Hirz M, Gärtner U, Jacquiet P, Taubert A, Hermosilla C (2020). *Besnoitia besnoiti* bradyzoite stages induce suicidal- and rapid vital-NETosis. Parasitology.

[123] Silva LMR, Lütjohann D, Hamid P, Velasquez ZD, Kerner K, Larrazabal C, Failing K, Hermosilla C, Taubert A (2019). *Besnoitia besnoiti* infection alters both endogenous cholesterol de novo synthesis and exogenous LDL uptake in host endothelial cells. Sci Rep.

[124] Hammoudeh N, Soukkarieh C, Murphy DJ, Hanano A (2020). Involvement of hepatic lipid droplets and their associated proteins in the detoxification of aflatoxin B1 in aflatoxin-resistance BALB/C mouse. Toxicol Rep.

[125] Kim TI, Kwon B, Yoon J, Park IJ, Bang GS, Park YK, Seo YS, Choi SY (2017). Antibacterial activities of graphene oxide-molybdenum disulfide nanocomposite films. ACS Appl Mater Interfaces.

[126] Yakimovich A, Georgi F. Induced cytopathic effects in live cells, 2018;3:1–14. 10.1128/mSphereDirect.00599-18 PMC6249643 30463927

[127] Bergaglio T, Synhaivska O, Nirmalraj PN (2024). 3D holo-tomographic mapping of COVID-19 microclots in blood to assess disease severity. Chem Biomed Imaging.

[128] Lee SY, Kim K, Mubarok A, Panduwirawan A, Lee KR, Lee S, Park HJ, Park YK (2014). High-resolution 3-D refractive index tomography and 2-D synthetic aperture imaging of live phytoplankton. J Opt Soc Korea.

[129] Umemura K, Matsukawa Y, Ide Y, Mayama S (2020). Label-free imaging and analysis of subcellular parts of a living diatom cylindrotheca sp. using optical diffraction tomography. Methods X..

[130] Jung JH, Hong SJ, Kim HB, Kim G, Lee M, Shin S, Lee SY, Kim DJ, Lee CG, Park YK (2018). Label-free non-invasive quantitative measurement of lipid contents in individual microalgal cells using refractive index tomography. Sci Rep.

[131] Singh AV, Laux P, Luch A, Sudrik C, Wiehr S, Wild AM, Santomauro G, Bill J, Sitti M (2019). Review of emerging concepts in nanotoxicology: opportunities and challenges for safer nanomaterial design. Toxicol Mech Methods.

[132] Bondarenko O, Mortimer M, Kahru A, Feliu N, Javed I, Kakinen A, Lin S, Xia T, Song Y, Davis TP, Lynch I, Parak WJ, Leong DT, Ke PC, Chen C, Zhao Y (2021). Nanotoxicology and nanomedicine: The Yin and Yang of nano-bio interactions for the new decade. Nano Today.

[133] Singh AV, Shelar A, Rai M, Laux P, Thakur M, Dosnkyi I, Santomauro G, Singh AK, Luch A, Patil R, Bill J. Harmonization risks and rewards: nano-QSAR for agricultural nanomaterials. 2024. 10.1021/acs.jafc.3c06466 10.1021/acs.jafc.3c06466 38315814

[134] Egbuna C, Parmar VK, Jeevanandam J, Ezzat SM, Patrick-Iwuanyanwu KC, Adetunji CO, Khan J, Onyeike EN, Uche CZ, Akram M, Ibrahim MS, El Mahdy NM, Awuchi CG, Saravanan K, Tijjani H, Odoh UE, Messaoudi M, Ifemeje JC, Olisah MC, Ezeofor NJ, Chikwendu CJ, Ibeabuchi CG (2021). Toxicity of nanoparticles in biomedical application: nanotoxicology. J Toxicol.

[135] Maiorano G, Sabella S, Sorce B, Brunetti V, Malvindi MA, Cingolani R, Pompa PP (2010). Effects of cell culture media on the dynamic formation of protein-nanoparticle complexes and influence on the cellular response. ACS Nano.

[136] Alkilany AM, Mahmoud NN, Hashemi F, Hajipour MJ, Farvadi F, Mahmoudi M (2016). Misinterpretation in nanotoxicology: a personal perspective. Chem Res Toxicol.

[137] Taka AL, Tata CM, Klink MJ, Mbianda XY, Mtunzi FM, Naidoo EB (2021). A review on conventional and advanced methods for nanotoxicology evaluation of engineered nanomaterials. Molecules.

[138] Friedrich RP, Schreiber E, Tietze R, Yang H, Pilarsky C, Alexiou C (2020). Intracellular quantification and localization of label-free iron oxide nanoparticles by holotomographic microscopy. Nanotechnol Sci Appl.

[139] Franqui LS, De Farias MA, Portugal RV, Costa CAR, Domingues RR, Souza Filho AG, Coluci VR, Leme AFP, Martinez DST (2019). Interaction of graphene oxide with cell culture medium: evaluating the fetal bovine serum protein corona formation towards in vitro nanotoxicity assessment and nanobiointeractions. Mater Sci Eng C.

[140] Zhou P, Guo M, Cui X (2021). Effect of food on orally-ingested titanium dioxide and zinc oxide nanoparticle behaviors in simulated digestive tract. Chemosphere.

[141] Batasheva S, Fakhrullina G, Akhatova F, Fakhrullin R. Caenorhabditis elegans nematode: a versatile model to evaluate the toxicity of nanomaterials in vivo. In: Nanotechnology characterization tools for environment, health, and safety; 2019. pp. 323–345. 10.1007/978-3-662-59600-5_11

[142] Jiang X, Lu C, Tang M, Yang Z, Jia W, Ma Y, Jia P, Pei D, Wang H (2018). Nanotoxicity of silver nanoparticles on HEK293T cells: a combined study using biomechanical and biological techniques. ACS Omega.

[143] Géloën A, Isaieva K, Isaiev M, Levinson O, Berger E, Lysenko V (2021). Intracellular detection and localization of nanoparticles by refractive index measurement. Sensors.

[144] Liu G, Gao J, Ai H, Chen X (2013). Applications and potential toxicity of magnetic iron oxide nanoparticles. Small.

[145] Khanal D, Zhang F, Song Y, Hau H, Gautam A, Yamaguchi S, Uertz J, Mills S, Kondyurin A, Knowles JC, Georgiou G, Ramzan I, Cai W, Ng KW, Chrzanowski W (2019). Biological impact of nanodiamond particles–label free, high-resolution methods for nanotoxicity assessment. Nanotoxicology.

[146] Roshanzadeh A, Park S, Ganjbakhsh SE, Park J, Lee DH, Lee S, Kim ES (2020). Surface charge-dependent cytotoxicity of plastic nanoparticles in alveolar cells under cyclic stretches. Nano Lett.

[147] Zapor L, Chojnacka-Puchta L, Sawicka D, Miranowicz-Dzierzawska K, Skowron J (2022). Cytotoxic and pro—in fl ammatory e ff ects of molybdenum and tungsten disulphide on human bronchial cells. Nanotechnol Rev.

[148] Suematsu Y, Tsai YA, Takeoka S, Franz CM, Arai S, Fujie T (2020). Ultra-thin, transparent, porous substrates as 3D culture scaffolds for engineering ASC spheroids for high-magnification imaging. J Mater Chem B.

[149] Kim S, Kang SH, Byun SH, Kim HJ, Park IK, Hirschberg H, Hong SJ (2020). Intercellular bioimaging and biodistribution of gold nanoparticle-loaded macrophages for targeted drug delivery. Electron.

[150] Kang SH, Shin YS, Lee DH, Park IS, Kim SK, Ryu D, Park Y, Byun SH, Choi JH, Hong SJ (2022). Interactions of nanoparticles with macrophages and feasibility of drug delivery for asthma. Int J Mol Sci.

[151] Sardarabadi H, Chafai DE, Gheybi F, Sasanpour P, Rafii-Tabar H, Cifra M (2020). Enhancement of the biological autoluminescence by mito-liposomal gold nanoparticle nanocarriers. J Photochem Photobiol B Biol.

[152] Singh AV, Bansod G, Mahajan M, Dietrich P, Singh SP, Rav K, Thissen A, Bharde AM, Rothenstein D, Kulkarni S, Bill J (2023). Digital transformation in toxicology: improving communication and efficiency in risk assessment. ACS Omega.

